# Clinical Outcomes and Safety of Spatially Fractionated Radiotherapy for Malignant Tumors: A Systematic Review and Evidence Map

**DOI:** 10.7759/cureus.118441

**Published:** 2026-10-23

**Authors:** Nandan M Shanbhag, Abdulrahman Bin Sumaida, Khalid S Balaraj

**Affiliations:** 1 Radiation Oncology/Palliative Care, Tawam Hospital, Al Ain, ARE; 2 Oncology/Radiation Oncology, Tawam Hospital, Al Ain, ARE

**Keywords:** grid, lattice, partial-tumor irradiation, radiation toxicity, spatially fractionated radiotherapy, systematic review

## Abstract

Spatially fractionated radiotherapy (SFRT) concentrates high doses in selected tumor regions while delivering lower doses between them. This systematic review evaluated clinical outcomes, treatment delivery, and harms across SFRT techniques, with particular attention to comparative evidence. PubMed, Europe PMC, and ClinicalTrials.gov were last searched on September 19, 2026; CINAHL, CENTRAL, Scopus, and WHO ICTRP were last searched on August 7, 2026. The original searches and selection were conducted by the corresponding author, with substantive artificial intelligence (AI) assistance and human checks across review stages. The September extension introduced further AI-assisted source verification and analysis. Eligible reports described patients receiving intentional SFRT for malignant tumors. Results were grouped by clinical question and synthesized without meta-analysis. Appraisal used the Cochrane risk-of-bias tool for randomized trials (RoB 2), Risk of Bias in Non-randomized Studies-of Interventions (ROBINS-I), and design-matched Joanna Briggs Institute (JBI) tools; selected comparative questions underwent Grading of Recommendations Assessment, Development and Evaluation (GRADE) evaluation. The final corpus comprised 151 reports representing 137 investigations. Evidence was predominantly noncomparative, with heterogeneous treatments, outcome definitions, and follow-up. A prematurely terminated randomized trial documented one response among nine participants assigned to GRID plus standard radiotherapy, with one unknown outcome, and one among three assigned to standard radiotherapy. A nonrandomized study in bulky unresectable non-small-cell lung cancer reported responses in 19/20 PATHY participants versus 4/20 receiving conventional palliative radiotherapy, but prognosis-based allocation prevented causal interpretation. Certainty was very low for four defined comparative questions. Several cohorts reported early tumor shrinkage or symptom improvement, while prospective evidence also showed nonuniform pain changes, progression, and relapse after initial response. Severe complications included obstruction, late genitourinary injury, and fatal carotid hemorrhage. Differences in endpoints, treatment combinations, analysis units, missingness, and possible cohort overlap precluded a defensible pooled effect or unique participant total. Restrictions by source access, design, era, and overlap preserved descriptive evidence of activity and delivery but did not establish comparative superiority, durable benefit, or general safety. SFRT warrants indication-specific evaluation with defined patient-important outcomes, reproducible dosimetry, and complete toxicity follow-up. The protocol and subsequent robustness amendment were retrospective and unregistered. No specific funding was received.

## Introduction and background

Bulky malignant tumors pose a challenge for radiotherapy planning because dose escalation across the entire target volume may exceed the tolerance of surrounding normal tissues. Spatially fractionated radiotherapy (SFRT) seeks to address this limitation by delivering high doses to selected regions within the tumor while maintaining lower doses in the intervening regions. GRID radiotherapy generates an array of radiation beamlets using a physical grid block or a multileaf collimator, whereas lattice radiotherapy creates discrete high-dose regions, termed vertices, arranged in three dimensions within the tumor. Particle-based SFRT, minibeam radiotherapy, and selected partial-tumor irradiation approaches use related principles but differ in their spatial configurations and dose prescriptions [1-3]. Whether these approaches improve the clinical therapeutic ratio compared with conventional radiotherapy remains uncertain.

The relevant benefit depends on treatment intent. For a patient with painful, bleeding, or compressive disease and limited life expectancy, rapid symptom relief with an acceptable treatment burden may be valuable even without longer survival. Preoperative treatment instead requires evidence about resectability, pathological response, and surgical morbidity. Definitive dose escalation requires durable tumor control and adequate observation of late injury. These distinctions matter because SFRT is frequently followed by whole-tumor irradiation, systemic treatment, or surgery, making outcomes after the complete pathway difficult to attribute to spatial fractionation alone [4].

Synthesizing the clinical literature presents additional challenges. Reports span historical grid-filter treatments and modern image-guided techniques, with evidence ranging from individual case reports to institutional cohorts and early clinical trials. Abstracts, full publications, registry results, and extended follow-up reports may describe overlapping patient populations. Radiological response may be reported per patient, lesion, or treatment course, often with analyses restricted to patients who survive and remain well enough for assessment. A high response proportion among evaluable patients therefore does not necessarily reflect the probability of clinical benefit among all patients offered treatment.

This systematic review evaluated clinical outcomes and harms across SFRT techniques, distinguishing comparative treatment effects from descriptive findings and assessing whether quantitative synthesis was justified.

## Review

Methodology

Protocol and Eligibility

Reporting followed the applicable recommendations of the Preferred Reporting Items for Systematic Reviews and Meta-Analyses (PRISMA) 2020 statement, its literature search extension (PRISMA-S), and the Synthesis Without Meta-analysis (SWiM) guideline [5-7]. The review protocol was formalized retrospectively on July 14, 2026, and was not registered. Amendments made on August 7 and 8, 2026, expanded the information sources and specified targeted retrieval procedures. A further unregistered amendment, dated September 19, 2026, was developed after the earlier review results were available. This amendment specified supplementary searches, source verification, focused critical appraisal, assessment of the certainty of evidence, structured synthesis, and restricted analyses examining the influence of source accessibility, study design, treatment era, and potential overlap between patient populations. The original protocol and subsequent amendments are reproduced in sections S5-S6 of the Supplementary Appendix; focused appraisal and restricted analyses are reported in the main article. A dated comparison of the protocol, amendments, and final methods, including the rationale for each change and its timing relative to the available findings, is provided in Supplementary Table S6. All post hoc analyses were exploratory and were not interpreted as confirmatory evidence.

The broad eligibility definition was used to map clinical experience with deliberately heterogeneous intratumoral irradiation across treatment eras and delivery platforms. Inclusion within this evidence map does not imply that techniques are biologically equivalent or have comparable clinical effects. The distinction between dose geometry and beam type, and the accompanying treatment pathway, is therefore retained in clinical interpretation [1-4].

Eligible reports described at least one patient treated for a tumor classified as malignant in the source report. Treatment had to involve an intentionally heterogeneous intratumoral dose distribution comprising high-dose and low-dose regions. Eligible techniques included GRID radiotherapy, lattice radiotherapy, particle-based SFRT, clinical minibeam radiotherapy, stereotactic central/core ablative radiation therapy (SCART), lattice extreme ablative dose (LEAD) radiotherapy, and partial-tumor irradiation explicitly based on an SFRT rationale. Conventional partial-volume irradiation without an explicit SFRT rationale was excluded. Randomized and nonrandomized comparative studies, prospective and retrospective cohorts, case series, and case reports were eligible. Original research abstracts and posted registry results were included when they provided clinical information on treated patients. Reviews, protocols without results, preclinical studies, and technical or simulation studies without eligible patient observations were excluded. No restrictions were imposed on language, publication year, tumor site, or comparator. Heterogeneity refers to the SFRT component, not necessarily to the entire course when conventional irradiation followed. An ordinary simultaneous integrated boost alone did not establish eligibility. Table 1 clarifies the definitions of eligible techniques.

**Table 1:** Definitions of eligible spatially fractionated radiotherapy techniques. Beam type, dose geometry, and named protocols can overlap. These definitions clarify the eligibility boundary and do not introduce new dose or geometric thresholds. SFRT: spatially fractionated radiotherapy; SCART: stereotactic central/core ablative radiation therapy; LEAD: lattice extreme ablative dose; PATHY: partial-tumor irradiation targeting a hypoxic segment

Technique	Operational definition	References
GRID radiotherapy	Patterned arrays of beamlets create high-dose regions separated by lower-dose regions	[1]
Lattice radiotherapy	Discrete high-dose vertices are arranged in three dimensions within the tumor, with lower doses between them	[2]
Particle-based SFRT	Proton or heavier-ion delivery implements an eligible spatially heterogeneous treatment pattern. Particle therapy alone does not establish eligibility	[8,9]
Clinical minibeam radiotherapy	Narrow, spatially separated beams produce alternating peak and valley doses in treated patients. Beam widths and spacing are retained as reported	[10]
SCART	Ablative irradiation of a central tumor core is combined with a lower dose at the tumor periphery	[11]
LEAD	A study-described lattice extreme ablative dose boost targets selected intratumoral subvolumes. Associated whole-target treatment is considered separately	[12]
Other partial-tumor approaches, including PATHY	Selected-subvolume irradiation is explicitly based on an SFRT rationale, such as targeting a source-defined hypoxic segment. Conventional partial-volume irradiation without that rationale remains excluded	[9,13]

Mixed-diagnosis cohorts were retained when they included eligible patients; outcomes that could not be separated for these patients were explicitly identified. Historical diagnoses were retained as reported and were not retrospectively reclassified in the absence of pathological evidence. A sensitivity analysis excluded reports in which eligibility under the malignancy criterion was uncertain and outcomes for patients with malignant tumors could not be established separately. When a modeling study included clinical measurements, these were considered separately from its simulated predictions.

Search Strategy, Study Selection, and Review Responsibilities

PubMed, Europe PMC, CINAHL Complete, the Cochrane Central Register of Controlled Trials (CENTRAL), Scopus, ClinicalTrials.gov, and the World Health Organization International Clinical Trials Registry Platform (WHO ICTRP) were searched through August 7, 2026. Search strategies included SFRT terminology and terms for GRID and lattice radiotherapy, particle-based treatments, minibeams, and named partial-tumor irradiation approaches. Targeted retrieval continued through August 9, 2026. A supplementary Google Scholar search retrieved 20 duplicate records before an access challenge prevented further searching. The search strategies were checked internally but did not undergo independent peer review using the Peer Review of Electronic Search Strategies (PRESS) framework [14].

On September 19, 2026, searches were repeated in PubMed, Europe PMC, and ClinicalTrials.gov. Searches in CINAHL Complete, CENTRAL, Scopus, and WHO ICTRP were not repeated. Supplementary citation searching and targeted searches of publisher websites, trial registries, and legally accessible repositories were undertaken to identify additional reports and more complete versions of previously identified reports. Duplicate publications and previously screened records were linked to existing review records before further eligibility decisions were made. Complete search procedures, exported candidate records, retrieval outcomes, and exclusions are provided in sections S1-S2 and S6 of the Supplementary Appendix. Authors of primary studies were not contacted.

The first author conducted the searches and initial study selection. The second and third authors independently checked study selection, full-text eligibility, extracted data, and risk-of-bias assessments, including the September 2026 additions. Disagreements between the checking authors were resolved through review by the first author.

Artificial intelligence (AI) tools assisted with screening, data extraction, critical appraisal, evidence synthesis, verification, and manuscript preparation. AI tools are not authors; the human authors are responsible for the content approved for submission. The complete report-level extraction and historical item-level appraisal are held in the authors’ archived review package and are available from the corresponding author on reasonable request.

Linkage, Extraction, and Source Verification

A report was defined as a publication or posted result, whereas an investigation referred to the underlying study. Companion reports were linked using trial identifiers, institutions, accrual periods, population characteristics, treatment details, and explicit cohort descriptions. Linked reports contributed complementary information to a single investigation. Distinct investigations with possible participant overlap were grouped into clusters without assuming that overlap had occurred.

Data extraction covered the study population, design, treatment intent, dose prescription and spatial geometry, co-interventions, follow-up, outcome definitions, analysis populations, and harms.

Radiological response required a reported result describing tumor size or imaging response. Pathological response, biochemical failure, local recurrence, symptom relief, functional status, and quality of life were separate constructs, retained in source terminology and interpreted within their own definitions. A populated extraction field, stated objective, or planned registry endpoint did not establish that an outcome was measured or analyzed.

Each result retains its value, analysis unit, denominator, time point, and source. Patient, lesion, course, and event units are not interchangeable. A composite endpoint, such as tumor regression or symptom relief, is not presented as either component alone. Rounded percentages were not converted to event counts without an exact compatible numerator and denominator.

Toxicity retains its reported scale, attribution, and time frame. Cumulative reporting through six months does not show that events began late, and a late-onset event is distinct from an event observed during longer follow-up. Nonreporting and missing follow-up were not treated as zero events.

Aggregate counts of studies reporting each outcome are not presented, because the underlying classifications cannot be applied uniformly across source formats. Study-specific results, including uncertain and nonseparable outcomes, remain available for inspection.

Reporting completeness is distinct from risk of bias in the underlying study. Full articles, conference abstracts, abstract-only views of articles, and registry results carry different amounts of information, and absence of a field from accessible material does not show that it was omitted from the complete publication. Funding and competing-interest statements may appear outside the main text; explicit statements of no funding or no competing interests were counted as disclosures. Publication-wide completeness percentages are therefore not presented, and source-specific extraction describes the information available for clinical interpretation.

For technical interpretation, the relevant fields are peak or vertex dose, valley or peripheral dose, geometry, target coverage, treatment position image guidance, organ-at-risk exposure, and the relationship to any subsequent whole-tumor irradiation. Imaging used for planning or response follow-up is not evidence of treatment position image guidance.

During the September 2026 verification, key extracted data were checked against retained or newly retrieved primary source material. The source version, extracted value, denominator, assessment time point, and location within the source were recorded. Verification records for every baseline report distinguished verified fields from those supported only by an abstract or a retained summary and identified fields that remained unresolved. Original research abstracts, full-text preprints, final published articles, and registry results were distinguished. Discrepancies within or between sources were retained when they could not be resolved using the available material. Report inventories, linkage records, outcome definitions, source discrepancies, and update records are provided in the main article and section S6 of the Supplementary Appendix.

Risk of Bias and Certainty of Evidence

The revised Cochrane risk-of-bias tool for randomized trials (RoB 2) was applied to individual results from randomized trials, and the 2016 version of the Risk of Bias in Non-randomized Studies-of Interventions (ROBINS-I) tool was applied to selected results from nonrandomized comparative intervention studies. Descriptive reports were assessed using design-specific critical appraisal tools from JBI, formerly the Joanna Briggs Institute [15-18].

Focused reassessment covered tumor response in the small randomized GRID trial; postoperative recurrence in the historical rectal cancer trial; response, symptoms, and survival in the partial-tumor comparison; and pain, radiological response, and severe dermatitis in the comparison of Cerrobend-block and multileaf-collimator GRID radiotherapy. An additional timing comparison underwent outcome-specific appraisal. For nonrandomized assessments, the hypothetical randomized target trial and key baseline confounders were specified. JBI assessments were updated for newly retrieved or newly included reports using the available source material. These targeted updates did not constitute a complete reassessment of all investigations. JBI item-level judgments were not summed into overall quality scores.

The Grading of Recommendations Assessment, Development and Evaluation (GRADE) approach was used to assess the certainty of evidence for outcomes within four defined comparative questions, considering risk of bias, inconsistency, indirectness, imprecision, and publication bias [19]. For nonrandomized evidence, ROBINS-I judgments against a hypothetical randomized target trial informed downgrading for risk of bias; no additional downgrade was applied solely for nonrandomized study design. The absence of common assessment time points and unclear definitions of the magnitude or duration of symptom benefit were considered when judging whether the evidence addressed the clinical question. Evidence was not downgraded for inconsistency solely because it came from a single study. No overall certainty rating was assigned to SFRT, and no rating was assigned when the effect of interest could not be reliably defined. Domain-level judgments and supporting explanations are provided in the main article under Result-specific Appraisal and Certainty.

Evidence Synthesis and Sensitivity Analyses

The synthesis addressed six clinical questions: incremental benefit relative to a comparator, tumor response in the palliative setting, symptom relief, durable local control, severe harms, and treatment delivery. Survival was considered separately as a contextual outcome and was not treated as a measure of local control. Studies were grouped by treatment intent, technique, comparator, outcome, and assessment time point. Randomized and other direct comparative studies informed assessments of comparative treatment effects; prospective cohorts provided descriptive evidence of antitumor activity and safety; and selected cases provided clinical signals or technical observations. Results were not synthesized by counting studies with statistically significant or favorable findings. A report-by-claim matrix linked every report-investigation relationship to source fields, and an investigation-level matrix grouped definite companions. Entries distinguished context, observed results, uncertain or nonseparable results, and absence of usable information; they were not counts of favorable findings.

For this revision, the clinical presentation was reorganized post hoc by dose geometry, beam type, treatment intent, and the relationship to subsequent whole-target irradiation, consistent with transparent reporting of synthesis groupings [7]. These are descriptive, exploratory groupings, not prespecified subgroup-effect analyses. Beam type and geometry may overlap: proton GRID is a GRID geometry delivered with particles, while Particle-PATHY is a partial-tumor approach. Linked reports remain one investigation. A separate conventional course is distinguished from the lower-dose component of an SFRT plan; planned, delivered, and incompletely reported subsequent treatment are not conflated. Missing information is labeled as not reported, not as absence of treatment. The clinical table illustrates distinct pathways rather than providing an exhaustive stratified count of all investigations.

The feasibility of meta-analysis was assessed for 15 narrowly defined questions, considering the compatibility of populations, treatments, comparators, outcome definitions, units of analysis, and assessment windows, as well as the independence of study cohorts. These clinical and statistical criteria replaced the earlier requirement for a minimum of three studies. No group of studies met the criteria for pooling. For two studies with sufficient group-level event counts and denominators, unadjusted risk ratios (RRs) and risk differences with 95% confidence intervals (CIs) were calculated as descriptive within-study comparisons. CIs were calculated on the logarithmic scale for RRs and using the Newcombe-Wilson method for risk differences. These intervals reflect sampling uncertainty conditional on the reported data and statistical assumptions; they do not account for confounding or incomplete measurement. No median of heterogeneous response proportions, pooled participant count, or average of incompatible results was calculated.

Post hoc sensitivity analyses examined the effects of restricting the synthesis to data contributed by full reports, prospective or comparative clinical cohorts, or reports published from 2010 onward. Separate analyses either excluded all investigations belonging to clusters with possible participant overlap or retained one representative investigation per cluster. Representative selection prioritized source completeness, study design, whole-cohort rather than selected-case coverage, the most recent results publication year, and a stable identifier, without considering whether outcomes were favorable. Each restriction was applied separately. Registry outcomes were not classified as full-report evidence solely because a case report describing a subset of the cohort was available.

In a post hoc sensitivity analysis, we excluded the historical hemangioblastoma report and mixed benign-malignant Particle-PATHY results without separable malignant-only outcomes. The durability analysis used local control outcomes assessed at 12 months or later; survival, biochemical failure, and earlier local control were considered separately. Details are reported under Quantitative Compatibility and Restricted Syntheses.

Results

Selection and Characteristics

The August 2026 searches identified 11,201 source occurrences, yielding 6,870 unique records after deduplication. Of the 232 reports sought for retrieval, 161 were assessed for eligibility, and 140 were included, representing 127 investigations. In total, 71 reports were initially unavailable. Supplementary searches, citation assessment, and targeted retrieval in September 2026 identified 11 additional eligible reports representing 10 additional investigations. The combined evidence base comprised 151 reports and 137 investigations (Figure 1).

**Figure 1:**
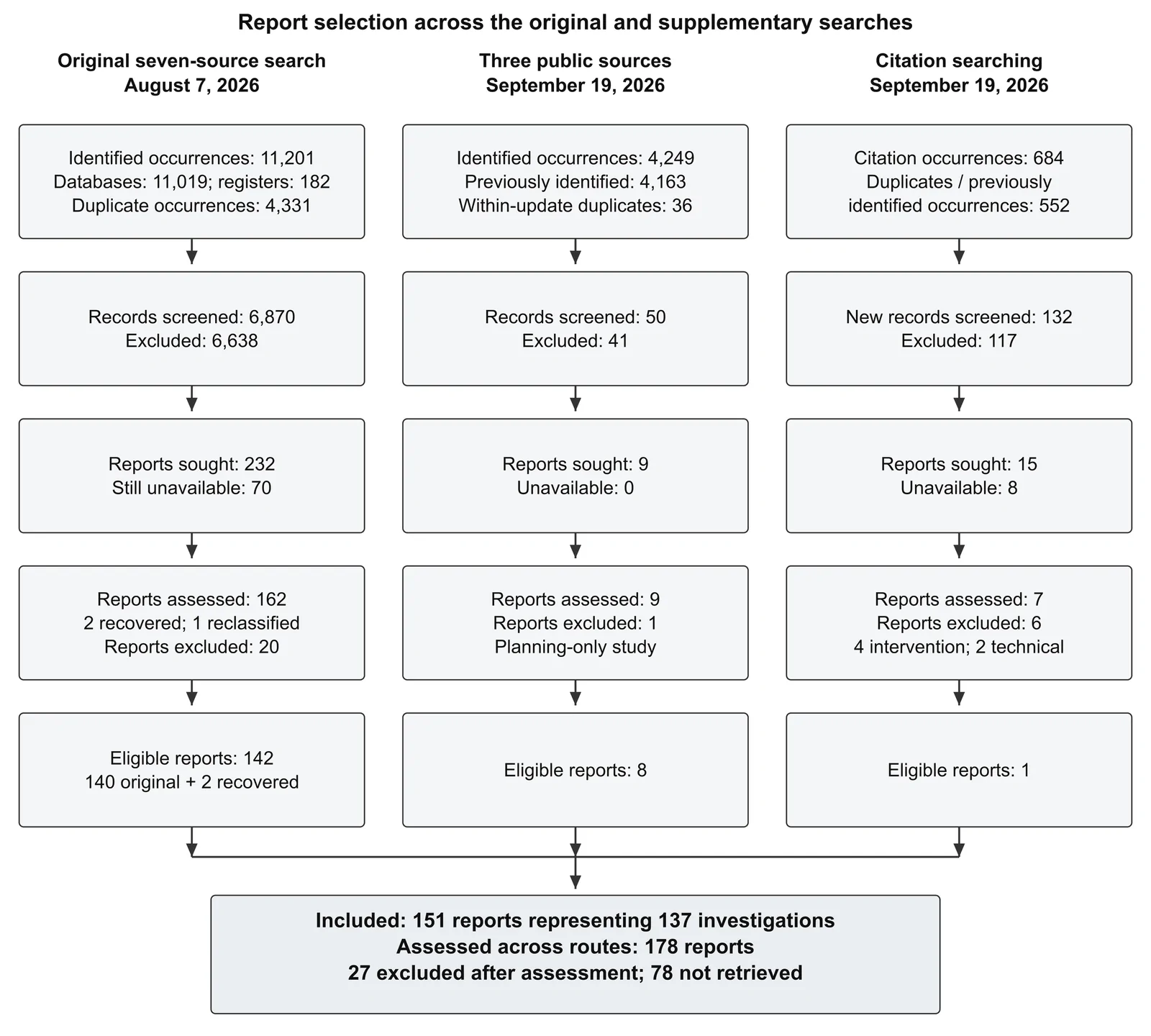
Selection of reports and investigations. The original seven-source search ended August 7, 2026. The September 19 extension searched three public sources and added citation assessment and targeted retrieval. Source-access upgrades for already included reports did not add investigations. One historical exclusion was moved to not retrieved after source reassessment. Companion reports and possible cohort overlap do not add independent participants. Citation duplicates include four manually reconciled identities and two public update matches.

Across all search and retrieval routes, 256 reports were sought, 178 were assessed for eligibility, and 27 were excluded after assessment; 78 reports remained unavailable. These totals account for the reclassification of one historical report from excluded to not retrieved because only its introductory text was accessible. Updated inventories of excluded and unavailable reports are provided in sections S3-S4 and S6 of the Supplementary Appendix.

Historical GRID treatments and early modern SFRT reports contributed to the evidence map [20-35]. A related LITE-SABR preprint is discussed separately below [36]. The remaining literature included reports of photon- and proton-based SFRT, lattice radiotherapy, minibeam radiotherapy, PATHY, and SCART, with evidence drawn from registry results, cohort studies, and individual cases [8-13,37-165].

The source ledger classified 92 reports as full-text records, 53 as abstract-level records, and six as registry results. Two reports classified as full text were supported only by retained summaries during verification and were therefore excluded from the sensitivity analysis restricted to actual full-report contributions. The full-text category included separately identified preprints and was not limited to final peer-reviewed articles. Source verification also distinguished original author text from records supported only by summaries. GRID and lattice radiotherapy were the predominant techniques. Most evidence was noncomparative, and only two randomized comparisons were identified. Potential overlap between institutional cohorts, repeated treatment courses, and differences in reporting units precluded a reliable estimate of the total number of unique participants.

Some reports provided only limited clinical information. The BLaStM contribution comprised four participants selected because they had positive biopsies at the study endpoint, within an imaging analysis that also included 21 LEAD participants. This report did not provide a separately extractable outcome estimate for the BLaStM trial as a whole [20]. A newly included modeling report contributed measured tumor and lymphocyte trajectories from four patients; its simulated treatment comparisons did not provide observed estimates of clinical efficacy [21]. These reports broadened the range of clinical observations but did not provide independent comparative treatment-effect estimates.

Included Report Inventory

Table 2 lists all 151 included reports with report identifier, reference number, title, year, linked investigation, and the source format used at the review cutoff. It provides citation coverage for included reports not discussed individually in the main text, and identifiers are stable across the inventory, evidence files, and manuscript. Source format is distinct from publication type: “Abstract” covers conference abstracts and journal articles accessed only through their abstracts, and does not imply that a fuller version cannot become available. Posted registry results are separate reports, linked to publications only where the study relationship is supported. Totals: 92 full text, 53 abstract and 6 posted registry results; 137 investigations and 152 report investigation links.

**Table 2:** Included reports (n = 151).

Report	Reference	Title (year)	Linked investigation	Source
SCR-00003	[72]	A new approach to the roentgen therapy of cancer with the use of a grid (1950)	ST-00003	Full text
SCR-00006	[64]	Experience with grid therapy (1952)	ST-00006	Full text
SCR-00009	[73]	Roentgen therapy through a grid for advanced carcinoma (1953)	ST-00009	Full text
SCR-00011	[74]	Inoperable carcinoma of antrum of Highmore; report of a twenty-year survival after telecurietherapy and of a five year survival after roentgen-grid therapy (1954)	ST-00011	Full text
SCR-00014	[75]	Roentgen therapy of advanced carcinoma of the female genitalia with the use of a grid (1954)	ST-00014	Full text
SCR-00018	[76]	[Technics, procedures and indications in grid therapy of breast tumors] (1955)	ST-00018	Full text
SCR-00031	[31]	Irradiation of advanced cancer of the head and neck through a grid. I. Study of absorbed dose by observation of skin and mucosal reactions (1957)	ST-00031	Full text
SCR-00037	[65]	Etude dosimetrique et clinique de la radio therapie a travers grille (1958)	ST-00037	Full text
SCR-00057	[77]	Focused grid telecobalt therapy (1961)	ST-00057	Abstract
SCR-00097	[32]	[Radiation therapy of patients with inoperable cancer of the pancreas] (1976)	ST-00097	Abstract
SCR-00103	[78]	[Total contact grid irradiation and its clinical importance (author’s transl)] (1979)	ST-00103	Abstract
SCR-00106	[79]	[Gamma teletherapy of inoperable retroperitoneal tumors with nonuniform distribution of the dosage in the tissues] (1980)	ST-00106	Abstract
SCR-00110	[28]	[Treatment effectiveness and the radiation complications in patients with inoperable esophageal cancer undergoing rotation gamma therapy by open field and through a grid] (1980)	ST-00110	Abstract
SCR-00111	[80]	[Clinical trial of a variation of combined radiation therapy of recurrent rectal cancer in women] (1981)	ST-00111	Abstract
SCR-00122	[23]	[Combination treatment of rectal cancer using 2 variants of preoperative concentration gamma therapy] (1983)	ST-00122	Abstract
SCR-00141	[81]	[Radiation and chemoradiation therapy of malignant laryngopharyngeal tumors] (1985)	ST-00141	Abstract
SCR-00151	[82]	[Radiotherapy of cerebral metastases of breast cancer. Clinical experience with 67 patients] (1987)	ST-00151	Abstract
SCR-00156	[83]	[Results of radiation and combination therapy of glomus tumors] (1988)	ST-00156	Abstract
SCR-00176	[84]	Lack of late skin necrosis in man after high-dose irradiation using small field sizes: experiences of grid therapy (1990)	ST-00176	Abstract
SCR-00177	[85]	Palliative treatment of advanced cancer using multiple nonconfluent pencil beam radiation. A pilot study (1990)	ST-00177	Abstract
SCR-00283	[41]	High-dose spatially-fractionated radiation (GRID): a new paradigm in the management of advanced cancers (1999)	ST-KY-GRID-MIXED-1995-1998	Full text
SCR-00347	[86]	The impact of TNF-alpha induction on therapeutic efficacy following high dose spatially fractionated (GRID) radiation (2002)	ST-00347	Abstract
SCR-00414	[87]	Elevated sphingomyelinase activity and ceramide concentration in serum of patients undergoing high dose spatially fractionated radiation treatment: implications for endothelial apoptosis (2005)	ST-00414	Abstract
SCR-00451	[88]	Spatially fractionated GRID radiation treatment of advanced neck disease associated with head and neck cancer (2006)	ST-KY-GRID-HNC-1995-2002	Full text
SCR-00521	[22]	Study of Advanced Bulky Malignancies With Spatially Fractioned Radiation Therapy (2015)	ST-NCT00765570	Registry results
SCR-00583	[89]	Evaluation of spatially fractionated radiotherapy (GRID) and definitive chemoradiotherapy with curative intent for locally advanced squamous cell carcinoma of the head and neck: initial response rates and toxicity (2010)	ST-00583	Full text
SCR-00630	[57]	MRI-Guided Lattice Extreme Ablative Dose Radiotherapy For Prostate Cancer (2021)	ST-MIAMI-LEAD-PHASE1	Registry results
SCR-00676	[27]	High-dose spatially fractionated GRID radiation therapy (SFGRT): a comparison of treatment outcomes with Cerrobend vs. MLC SFGRT (2012)	ST-00676	Full text
SCR-00754	[68]	Safety of LEAD Radiotherapy Plus Chemoradiation in Patients With Bulky Stage III Non-Small Cell Lung Cancer (2017)	ST-NCT01797471	Registry results
SCR-00854	[90]	High-Dose Radiation as a Dramatic, Immunological Primer in Locally Advanced Melanoma (2015)	ST-00854	Full text
SCR-00902	[91]	The Use of Lattice Radiation Therapy (LRT) in the Treatment of Bulky Tumors: A Case Report of a Large Metastatic Mixed Mullerian Ovarian Tumor (2015)	ST-AMENDOLA-OVARIAN-CASE	Full text
SCR-01010	[92]	Hunting for abscopal and bystander effects: clinical exploitation of non-targeted effects induced by partial high-single-dose irradiation of the hypoxic tumour segment in oligometastatic patients (2017)	ST-KABEG-PATHY-INITIAL-7	Full text
SCR-01086	[93]	Improved outcome of treating locally advanced lung cancer with the use of Lattice Radiotherapy (LRT): A case report (2018)	ST-AMENDOLA-NSCLC-10	Full text
SCR-01110	[94]	Spatially fractionated (GRID) radiation therapy using proton pencil beam scanning (PBS): Feasibility study and clinical implementation (2018)	ST-CHICAGO-PROTON-GRID-4	Abstract
SCR-01135	[95]	Clinical Outcomes of Spatially Fractionated GRID Radiotherapy in the Treatment of Bulky Tumors of the Head and Neck (2019)	ST-01135	Full text
SCR-01169	[24]	Mono-institutional phase 2 study of innovative Stereotactic Body RadioTherapy targeting PArtial Tumor HYpoxic (SBRT-PATHY) clonogenic cells in unresectable bulky non-small cell lung cancer: profound non-targeted effects by sparing peri-tumoral immune microenvironment (2019)	ST-01169	Full text
SCR-01171	[13]	Novel stereotactic body radiation therapy (SBRT)-based partial tumor irradiation targeting hypoxic segment of bulky tumors (SBRT-PATHY): improvement of the radiotherapy outcome by exploiting the bystander and abscopal effects (2019)	ST-KABEG-PATHY-23	Full text
SCR-01172	[66]	Palliative Lattice Stereotactic Body Radiotherapy (SBRT) (2021)	ST-01517	Registry results
SCR-01181	[96]	Safety and Efficacy of Lattice Radiotherapy in Voluminous Non-small Cell Lung Cancer (2019)	ST-AMENDOLA-NSCLC-10	Full text
SCR-01198	[33]	Time-synchronized immune-guided SBRT partial bulky tumor irradiation targeting hypoxic segment while sparing the peritumoral immune microenvironment (2019)	ST-01198	Full text
SCR-01230	[97]	Combined High-Dose LATTICE Radiation Therapy and Immune Checkpoint Blockade for Advanced Bulky Tumors: The Concept and a Case Report (2020)	ST-01230	Full text
SCR-01255	[8]	Early clinical results of proton spatially fractionated GRID radiation therapy (SFGRT) (2020)	ST-CHICAGO-PROTON-GRID-10	Full text
SCR-01288	[44]	Palliative Lattice Stereotactic Body Radiotherapy (SBRT) for Patients With Sarcoma, Thoracic, Abdominal, and Pelvic Cancers (2023)	ST-NCT04553471	Registry results
SCR-01289	[12]	Phase I Trial of MRI-Guided Prostate Cancer Lattice Extreme Ablative Dose (LEAD) Boost Radiation Therapy (2020)	ST-MIAMI-LEAD-PHASE1	Full text
SCR-01303	[98]	Spatially Fractionated Radiation Therapy Using Lattice Radiation in Far-advanced Bulky Cervical Cancer: A Clinical and Molecular Imaging and Outcome Study (2020)	ST-AMENDOLA-CERVICAL-10	Abstract
SCR-01304	[53]	Spatially Fractionated Radiotherapy (GRID) Prior to Standard Neoadjuvant Conventionally Fractionated Radiotherapy for Bulky, High-Risk Soft Tissue and Osteosarcomas: Feasibility, Safety, and Promising Pathologic Response Rates (2020)	ST-MARYLAND-SARCOMA-GRID-26	Abstract
SCR-01363	[54]	Dosimetric Parameters Associated With Pathologic Response in Patients Undergoing Preoperative Spatially Fractionated Radiotherapy (GRID) for Soft Tissue Sarcoma (2021)	ST-MARYLAND-SARCOMA-GRID-26	Abstract
SCR-01389	[99]	Lattice Radiotherapy (LRT)-Spatially Fractionated Radiotherapy (SFRT): Advanced Non-Small Cell Lung Cancer (NSCLC): Early Experience (2021)	ST-01389	Abstract
SCR-01455	[100]	VMAT Grid Therapy: A Widely Applicable Planning Approach (2021)	ST-01455	Abstract
SCR-01462	[101]	A Novel Radiotherapeutic Approach to Treat Bulky Metastases Even From Cutaneous Squamous Cell Carcinoma: Its Rationale and a Look at the Reliability of the Linear-Quadratic Model to Explain Its Radiobiological Effects (2022)	ST-01462	Full text
SCR-01502	[102]	First-ever Clinical Experience With Magnetic Resonance-based Lattice Radiotherapy for Treating Bulky Gynecological Tumors (2022)	ST-01502	Abstract
SCR-01508	[103]	How a very large sarcomatoid lung cancer was efficiently managed with lattice radiation therapy: a case report (2022)	ST-01508	Abstract
SCR-01509	[104]	Impressive Results after “Metabolism-Guided” Lattice Irradiation in Patients Submitted to Palliative Radiation Therapy: Preliminary Results of LATTICE_01 Multicenter Study (2022)	ST-01509	Full text
SCR-01517	[67]	LITE SABR M1: A phase I trial of Lattice stereotactic body radiotherapy for large tumors (2022)	ST-01517	Full text
SCR-01519	[20]	Longitudinal Changes and Predictive Value of Multiparametric MRI Features for Prostate Cancer Patients Treated with MRI-Guided Lattice Extreme Ablative Dose (LEAD) Boost Radiotherapy (2022)	ST-MIAMI-BLASTM; ST-MIAMI-LEAD-PHASE1	Full text
SCR-01522	[105]	Magnetic Resonance Imaging-Guided Online Adaptive Lattice Stereotactic Body Radiotherapy in Voluminous Liver Metastasis: Two Case Reports (2022)	ST-01522	Full text
SCR-01536	[9]	Novel Carbon Ion and Proton Partial Irradiation of Recurrent Unresectable Bulky Tumors (Particle-PATHY): Early Indication of Effectiveness and Safety (2022)	ST-01536	Full text
SCR-01541	[106]	Physical aspects of a spatially fractionated radiotherapy technique for large soft tissue sarcomas (2022)	ST-BORZOV-ISRAEL-LRT-SARCOMA-3	Full text
SCR-01571	[107]	Tumor Lysis Syndrome in a Patient With Metastatic Endometrial Cancer Treated With Lattice Stereotactic Body Radiation Therapy (2022)	ST-NCT04553471	Full text
SCR-01575	[108]	A Case Report on Initial Experience of Lattice Radiation Therapy for Managing Massive Non-small Cell Lung Cancer and Renal Pelvic Cancer (2023)	ST-01575	Full text
SCR-01611	[109]	First treatments for Lattice stereotactic body radiation therapy using magnetic resonance image guided radiation therapy (2023)	ST-01611	Full text
SCR-01638	[110]	Neoadjuvant Radiation Therapy with Interdigitated High-Dose LRT for Voluminous High-Grade Soft-Tissue Sarcoma (2023)	ST-01638	Full text
SCR-01661	[111]	Salvage LATTICE radiotherapy for a growing tumour despite conventional radio chemotherapy treatment of lung cancer (2023)	ST-01661	Full text
SCR-01741	[112]	Effectiveness of radiotherapy in delaying treatment changes in primary or secondary immunorefractory oligoprogressive patients: preliminary results from a single-center study (2024)	ST-01741	Full text
SCR-01774	[113]	Lattice radiotherapy in inflammatory breast cancer: report of a first case treated with curative aim (2024)	ST-01774	Full text
SCR-01783	[10]	Minibeam Radiation Therapy Treatment (MBRT): Commissioning and First Clinical Implementation (2024)	ST-MAYO-MBRT-FIRST-2	Full text
SCR-01789	[29]	Novel time-synchronized immune-guided partial tumor irradiation: Proof of principle trial (2024)	ST-NCT04168320	Abstract
SCR-01814	[50]	Spatially Fractionated Radiation Therapy in Sarcomas: A Large Single-Institution Experience (2024)	ST-MAYO-SFRT-REGISTRY-2019-2022	Full text
SCR-01818	[114]	Spatially fractionated radiotherapy (Lattice SFRT) in the palliative treatment of locally advanced bulky unresectable head and neck cancer (2024)	ST-01818	Full text
SCR-01823	[11]	Stereotactic central/core ablative radiation therapy: results of a phase I study of a novel strategy to treat bulky tumor (2024)	ST-SCART-PHASE1	Full text
SCR-01824	[59]	Stereotactic Core Ablative Radiation Therapy (SCART) - principal and practice of a novel strategy to treat bulky tumor (2024)	ST-SCART-PHASE1	Full text
SCR-01833	[40]	The Promising Effects of Lattice Radiotherapy for Large, Fungating, or Ulcerating Breast Cancers: A Prospective Single-center Study (2024)	ST-01833	Full text
SCR-01838	[37]	Time-Related Outcome Following Palliative Spatially Fractionated Stereotactic Radiation Therapy (Lattice) of Large Tumors - A Case Series (2024)	ST-LUCERNE-LATTICE-PALLIATIVE	Full text
SCR-01865	[115]	Advancing Intracranial Tumor Treatment Through Gyroscopic Radiosurgery-Based Lattice Therapy: Evidence From a Case Series Study (2025)	ST-01865	Full text
SCR-01879	[116]	Bulky non-small cell lung cancer treated with two-field robust proton lattice boost followed by conventional chemoradiation (2025)	ST-01879	Full text
SCR-01883	[117]	Case Report: Complete pathologic response in advanced melanoma with SFRT and dual checkpoint inhibition (2025)	ST-01883	Full text
SCR-01884	[118]	Case Report: Early experience with hypoxia-targeted SBRT-based partial tumor irradiation in large and bulky tumors (2025)	ST-01884	Full text
SCR-01885	[119]	Case Report: Navigating novel therapies and systemic barriers in ovarian clear cell carcinoma through a complete response to lenvatinib, pembrolizumab, and SFRT (2025)	ST-01885	Full text
SCR-01902	[38]	Diagnosis-Related Outcome Following Palliative Spatially Fractionated Radiation Therapy (Lattice) of Large Tumors (2025)	ST-LUCERNE-LATTICE-PALLIATIVE	Full text
SCR-01928	[120]	Experience With the Lattice Technique of Palliative Radiotherapy for Treating Voluminous Metastases of Renal Cancer in the Radiotherapy Department of Solca Quito, Ecuador: A Case Report (2025)	ST-01928	Full text
SCR-01942	[121]	Highs and Lows of Spatially Fractionated Radiation Therapy: Dosimetry and Clinical Outcomes (2025)	ST-MAYO-SFRT-REGISTRY-2019-2022	Abstract
SCR-01947	[122]	Initial experience of spatially fractionated lattice radiation therapy for palliative treatment of pediatric bulky tumors (2025)	ST-01947	Full text
SCR-01951	[39]	Interim Analysis of Pro-Grid: A Phase 1 Proton Spatially Fractionated Radiation Therapy Trial (2025)	ST-01951	Full text
SCR-01958	[123]	Lattice radiotherapy for extensive liver metastases in synchronous EGFR-mutant lung adenocarcinoma and small cell lung cancer (2025)	ST-01958	Full text
SCR-01966	[124]	Metabolism-Guided LATTICE Radiotherapy in an Elderly Patient with Locally Advanced Head and Neck Cancer Treated with Curative Aim: A Case Report (2025)	ST-01966	Full text
SCR-01968	[125]	Metastatic colon adenocarcinoma to the gingiva treated with spatial fractionation radiotherapy: a case report (2025)	ST-01968	Full text
SCR-01972	[126]	Mini-lattice radiation therapy: A treatment planning approach to miniaturize spatially fractionated lattice radiation therapy using a clinical linear accelerator (2025)	ST-MAYO-MLRT-FIRST-PATIENT	Abstract
SCR-01974	[127]	Minibeam Radiation Therapy for Recurrent Mucosal Melanoma: An Eye-Opening Response (2025)	ST-MAYO-MBRT-MELANOMA	Full text
SCR-01987	[128]	Novel Gradient-based Approach to Spatially Fractionated Radiotherapy: Implementation and Preliminary Clinical Results (2025)	ST-01987	Full text
SCR-02008	[129]	Plan evaluation tool for spatially fractionated radiation therapy for unresectable large tumors via spatial biological effective dose modeling in combination therapy (2025)	ST-KY-MLC-HNC-SBED-10	Abstract
SCR-02014	[130]	Primary Hepatic Neuroendocrine Tumor Treated With Lattice and External Beam Radiation: A Case Report (2025)	ST-02014	Full text
SCR-02031	[131]	Reported Early Clinical Outcomes of Forward-Planned Multileaf Collimator-Based 3-Dimensional Conformal Spatially Fractionated Radiation Therapy Technique for Large and Bulky Tumors (2025)	ST-KY-MLC-MIXED-23	Abstract
SCR-02035	[52]	Role of Spatially Fractionated Radiotherapy (LATTICE) Treatment in Inoperable Bulky Soft-Tissue Sarcomas (2025)	ST-02035	Full text
SCR-02038	[132]	SFRT combined with immunotherapy for a growing hepatocellular carcinoma after the failure of anti-angiogenesis and anti-PD1 treatment: a case report (2025)	ST-02038	Full text
SCR-02047	[42]	Spatially Fractionated Radiation Therapy for Palliation in Patients With Large Cancers: A Retrospective Study (2025)	ST-02047	Full text
SCR-02049	[133]	Spatially fractionated radiotherapy combined with CapeOX and bevacizumab for postoperative pulmonary metastasis from urachal carcinoma: a case report (2025)	ST-02049	Full text
SCR-02100	[134]	Case Report: Innovative radiotherapy strategies in advanced colon cancer with oligometastasis: a case of online adaptive radiotherapy and lattice stereotactic re-irradiation (2026)	ST-02100	Full text
SCR-02101	[135]	Case Report: Spatially fractionated radiation therapy for local recurrence after prior radiotherapy in advanced right-sided small cell lung cancer with mediastinal lymph node metastases (2026)	ST-02101	Full text
SCR-02102	[136]	Case Report: Three-fraction lattice radiotherapy followed by VMAT concurrent chemoradiotherapy in locally advanced non-small cell lung cancer (2026)	ST-02102	Full text
SCR-02127	[62]	Encouraging Local Control With Pediatric Spatially Fractionated Radiation Therapy (2026)	ST-MAYO-PEDS-SFRT-5	Full text
SCR-02136	[137]	Feasibility, safety, and clinical outcomes analysis of dense-lattice radiation therapy in palliative care for cancer patients (2026)	ST-02136	Abstract
SCR-02139	[63]	Grid Spatially Fractionated Radiation Therapy for Bulky Tumors: A Large Single Institution Experience (2026)	ST-02139	Full text
SCR-02162	[138]	Lattice Radiation Therapy as a Novel Approach for Renal Cell Carcinoma: A Case Series (2026)	ST-02162	Full text
SCR-02164	[139]	Lattice Radiation Therapy Plays a Synergistic Role in the Radical Treatment of Bulky Cervical Cancer: A Case Report and Literature Review (2026)	ST-02164	Full text
SCR-02168	[140]	Lattice radiotherapy for palliation in a case of metastatic adrenocortical carcinoma (2026)	ST-02168	Abstract
SCR-02192	[141]	Preliminary results from a phase II trial of spatially fractionated radiotherapy combined with immunotherapy and anti-angiogenic therapy in patients with bulky solid tumors: early evidence of promising efficacy and favorable safety (2026)	ST-02192	Full text
SCR-02202	[142]	Rapid Hemostasis and Significant Tumor Regression in Ulcerative Breast Cancer Via Combined Modified Lattice Radiotherapy and Systemic Therapy: A Case Report (2026)	ST-02202	Full text
SCR-02212	[143]	SCART sequential lattice spatial fractionated radiotherapy combined with chemotherapy for recurrent ovarian cancer with bulky bone metastasis: a case report (2026)	ST-SCART-OVARIAN-CASE-2025	Full text
SCR-02214	[144]	SFRT combined with immunotherapy for bulky hepatic metastasis from pancreatic acinar cell carcinoma: a case report (2026)	ST-02214	Full text
SCR-02223	[145]	Spatially fractionated radiotherapy for re-irradiation: feasibility, safety, treatment planning, and outcomes (2026)	ST-02223	Full text
SCR-02241	[69]	The Use of Spatially Fractionated Radiation Therapy Combined With Immune Checkpoint Inhibitors and Vascular Endothelial Growth Factor Inhibitor in the Treatment of Giant Metastatic Hepatocellular Carcinoma: A Case Report (2026)	ST-02241	Full text
SCR-02250	[146]	Spatially Fractionated Radiotherapy (SFRT) for Large Tumors: A Single Institutional Experience of Dosimetry and Clinical Outcomes (2024)	ST-AMARELL-SFRT-2019-2023	Abstract
SCR-02252	[147]	The Use of Lattice Radiation Therapy in Patients With Voluminous Tumors (2020)	ST-AMENDOLA-LATTICE-56	Abstract
SCR-02255	[148]	Definitive GRID and Fractionated Radiation in Bulky Head and Neck Cancer Associated With Low Rates of Distant Metastasis (2015)	ST-KY-HNC-GRID-53	Abstract
SCR-02256	[149]	Lattice radiotherapy-induced dramatic regression of bulky metastases challenges radioresistance compared with homogeneous hypofractionation in anorectal mucosal melanoma: a case report (2026)	ST-FERINI-AMM-LATTICE-CASE	Full text
SCR-02265	[150]	High-Dose Spatially Fractionated Radiotherapy, Lattice Radiotherapy in Bulky Tumors: Results after One Year Follow-Up (2022)	ST-LARREA-LATTICE-21	Abstract
SCR-02269	[43]	Re-Treatment of Recurrent Bulky Lesions with High Single Dose Partial Irradiation Targeting the Hypoxic Tumor Segment (PITH): A Case Series (2019)	ST-MASSACCESI-PITH-8	Abstract
SCR-02273	[151]	Spatially fractionated (GRID) radiation for palliative treatment of advanced cancer (1996)	ST-MOHIUDDIN-PALLIATIVE-GRID-61	Abstract
SCR-02275	[152]	Clinical Experience with Palliative Treatment of Bulky Tumors Using VMAT-Based Lattice Radiotherapy (2024)	ST-RAIDEN-LATTICE-63	Abstract
SCR-02278	[153]	Management of bulky high-grade pleomorphic sarcoma using grid therapy technique (2021)	ST-TAJIKI-PLEOMORPHIC-SARCOMA-CASE	Full text
SCR-02287	[154]	Utilization of spatially fractionated radiotherapy in the management of a patient with inoperable uterine leiomyosarcoma (2024)	ST-SILLANPAA-UTERINE-LMS-CASE	Full text
SCR-02290	[70]	Spatially fractionated radiotherapy for liver metastases: two cases of intrahepatic response dissociation (2026)	ST-NCT07193641	Full text
SCR-02344	[155]	A Five-Year Institutional Experience With Spatially Fractionated Radiation Therapy for Palliative Management of Bulky Tumors Across Diverse Anatomical Sites (2026)	ST-CACEROS-EL-SALVADOR-SFRT-60	Full text
SCR-02378	[156]	Phase I Clinical Trial of GRID Therapy in Pediatric Osteosarcoma of the Extremity (2022)	ST-NCT03139318	Registry results
SCR-02588	[25]	SBRT-Partial Tumor Irradiation for The Treatment of Unresectable Bulky NSCLC (2020)	ST-01169	Abstract
SCR-02599	[51]	Spatially Fractionated Radiation Therapy in the Modern Era: The Mayo Clinic Experience (2022)	ST-MAYO-SFRT-ABSTRACT-126	Abstract
SCR-02603	[60]	The Safety and Efficacy of SCART for Bulky Metastatic or Recurrent Cancer, a Phase I Study (2023)	ST-SCART-PHASE1	Abstract
SCR-02604	[30]	Time-Synchronized Immune-Guided Partial Tumor Irradiation: Proof-of-Principle Trial (NCT04168320) (2024)	ST-NCT04168320	Abstract
SCR-02605	[157]	Promising Pathologic Response of Spatially Fractionated Radiotherapy Preceding Standard Neoadjuvant Conventionally Fractionated Radiotherapy in Aggressive Soft Tissue Sarcoma (2024)	ST-MARYLAND-SARCOMA-SFRT-37	Abstract
SCR-02606	[49]	Spatially Fractionated Radiation Therapy (SFRT) Based on Multimodal Imaging: A Promising Salvage Treatment Strategy for Metastatic and Refractory Cancer Patients (2024)	ST-GUO-MULTIMODAL-SFRT-16	Abstract
SCR-02617	[158]	P2.01-07 Safety and Efficacy of LATTICE Radiotherapy in Voluminous Non-Small Cell Lung Cancer: A Retrospective Study of 10 Patients Over 7 Year Period (2018)	ST-AMENDOLA-NSCLC-10	Abstract
SCR-02619	[26]	EP1.01-02 Novel Partial Tumor Irradiation Improves Treatment Outcomes for Inoperable Bulky Lung Cancer Compared with Standard of Care (2019)	ST-01169	Abstract
SCR-02778	[159]	Efficacy of VMAT-lattice Spatially Fractionated Radiation Therapy (SFRT) for the treatment of large abdominal sarcoma (2023)	ST-MONTERO-GASTRIC-LMS-CASE	Full text
SCR-02926	[34]	[Radiological and combined therapy of laryngeal neoplasm metastasis] (1976)	ST-MOROZOV-LARYNX-METASTASES-52	Abstract
SCR-03042	[160]	[Immediate results of radiation therapy of rectum carcinoma with non-uniform irradiation (author’s transl)] (1979)	ST-SARKISJAN-RECTAL-GRID-26	Abstract
SCR-03067	[161]	[Concentrated nonuniform irradiation of locally spread esophageal cancer] (1978)	ST-KHARITONOVA-OESOPHAGEAL-GRID-22	Abstract
SCR-03961	[162]	Effectiveness and safety of 3D lattice radiation therapy in the gluteal region to symptomatically control unresectable synovial sarcoma: A geriatric case report (2025)	ST-TAJAMMUL-SYNOVIAL-SARCOMA-CASE	Abstract
SCR-05905	[163]	[Innovation in radiotherapy of advanced malignant tumors] (1967)	ST-EGAWA-LUNG-METASTASIS-GRID-CASE	Abstract
SCR-06355	[164]	Locally advanced thymoma treated with lattice radiotherapy: a case report (2024)	ST-LIU-THYMOMA-LATTICE-CASE	Full text
SCR-06514	[165]	Hemangioblastoma Cerebelli: Report of a Six Year Survival After Roentgen Therapy by Tumor Oscillation Through a Grid (1963)	ST-06514	Full text
V3-NEW-002	[55]	Lattice SFRT in a geriatric patient with an initially inoperable leg bulky sarcoma: case report and literature review (2026)	ST-V3-002	Full text
V3-NEW-012	[45]	SFRT alleviates symptoms associated with bulky advanced cancer: a retrospective single-center cohort analysis (2026)	ST-V3-012	Full text
V3-NEW-022	[47]	Individualized VMAT lattice radiotherapy on a standard flattening-filter linear accelerator for advanced bulky tumors: a retrospective feasibility and early clinical experience (2026)	ST-V3-022	Full text
V3-NEW-023	[61]	VMAT-based lattice radiotherapy for advanced bulky hepatocellular carcinoma within multimodality treatment: a consecutive feasibility series (2026)	ST-V3-023	Full text
V3-NEW-030	[21]	Exploratory personalized radiobiological modeling of bystander and immune effects to inform SFRT-SBRT scheduling (2026)	ST-V3-030	Full text
V3-NEW-036	[46]	Clinical Outcomes of Minibeam Radiotherapy for Superficial Skin and Soft Tissue Malignancies (2026)	ST-V3-036	Abstract
V3-NEW-041	[56]	Grid Radiation Therapy Based on Multileaf Collimator for Neoadjuvant Treatment of Large-Volume Intrahepatic Cholangiocarcinoma: A Case Report (2026)	ST-V3-041	Full text
V3-NEW-042	[48]	Exploring the Limits of Radiation Dose Gradient Compression in Bulky Solid Tumors Invading the Spinal Cord: An Exploratory Study of Conventional Volumetric Modulated Arc Therapy versus LATTICE Radiotherapy on a 10-mm Multi-Leaf Collimator Platform (2026)	ST-V3-042	Full text
SCR-02274	[58]	Dosimetry and Toxicity of a Phase I Clinical Trial of Men Treated for Prostate Cancer with Lattice Extreme Ablative Dose (LEAD) Radiation Therapy (2018)	ST-MIAMI-LEAD-PHASE1	Abstract
SCR-02613	[71]	Prognostic Factors for Early Demise Following Palliative Spatially Fractionated Radiotherapy (2024)	ST-MAYO-EARLY-DEMISE-306	Abstract
CHASE-0219	[35]	PO-0886 Partial tumor irradiation exploiting immunomediated effects: tumor microenvironment as a new oar (2019)	ST-V3-CIT-0219-KABEG32	Abstract

Report-to-Study Linkage and Possible Overlap

Companion reports were linked only on explicit evidence: cohort statements, trial identifiers, institution, accrual dates, eligibility, sample size, and treatment details. Similar titles or shared authorship alone did not establish identity, and an updated publication enlarging a cohort was not treated as independent replication. Distinct studies with possible participant overlap remained separately listed and were assigned to 14 possible-overlap clusters (Table 3), which were examined in a separate restricted synthesis (see Quantitative Compatibility and Restricted Syntheses in the main article).

**Table 3:** Possible-overlap clusters (n = 14).

Cluster	Studies	Reason independence is uncertain
OC-AMENDOLA-LATTICE-2010-2020	ST-AMENDOLA-CERVICAL-10; ST-AMENDOLA-LATTICE-56; ST-AMENDOLA-NSCLC-10; ST-AMENDOLA-OVARIAN-CASE	The 56-patient program cohort spans the period of the earlier disease-specific reports; its abstract does not identify which earlier participants, if any, it contains
OC-CHICAGO-PROTON-GRID	ST-CHICAGO-PROTON-GRID-10; ST-CHICAGO-PROTON-GRID-4	The later 10-patient series may contain the first four implementation cases; no participant crosswalk is provided
OC-FERINI-LATTICE-2020-2026	ST-01509; ST-FERINI-AMM-LATTICE-CASE	The melanoma case shares investigators and program context with LATTICE_01, but enrollment in that cohort is not stated
OC-HIST-MARKS-NYC-GENITAL-GRID	ST-00003; ST-00014	Shared author, era, disease setting, and a vulvar observation; no patient crosswalk or containment statement
OC-KABEG-PATHY-2016-2024	ST-01169; ST-01198; ST-KABEG-PATHY-23; ST-KABEG-PATHY-INITIAL-7; ST-NCT04168320; ST-V3-CIT-0219-KABEG32	Shared investigators, program and overlapping accrual. NCT04168320 is retained as a distinct trial, but reuse of participants from earlier KABEG reports cannot be excluded
OC-KY-GRID-1990-2015	ST-00177; ST-KY-GRID-HNC-1995-2002; ST-KY-GRID-MIXED-1995-1998; ST-KY-HNC-GRID-53; ST-MOHIUDDIN-PALLIATIVE-GRID-61	Pilot, palliative, mixed-site, and head and neck reports from the Kentucky (Mohiuddin) program; shared investigators and overlapping eras are compatible with containment, but no crosswalk proves it
OC-KY-MLC-2019-2024	ST-KY-MLC-HNC-SBED-10; ST-KY-MLC-MIXED-23	Shared investigators and platform; nesting of the 10-patient head and neck analysis within the 23-patient mixed-site cohort is not established
OC-LARREA-LATTICE-2020-2022	ST-01389; ST-LARREA-LATTICE-21	The 21-patient cohort and the earlier 11-patient non-small-cell lung carcinoma report share investigators and accrual period; containment is not established
OC-MARYLAND-SARCOMA-GRID-2009-2024	ST-MARYLAND-SARCOMA-GRID-26; ST-MARYLAND-SARCOMA-SFRT-37	The 37-patient 2009–2023 report shares investigators, setting, population and regimen with the 26-patient reports; no crosswalk or explicit update statement
OC-MAYO-SFRT-PEDS-2019-2025	ST-MAYO-EARLY-DEMISE-306; ST-MAYO-MLRT-FIRST-PATIENT; ST-MAYO-PEDS-SFRT-5; ST-MAYO-SFRT-ABSTRACT-126; ST-MAYO-SFRT-REGISTRY-2019-2022	First-treatment report, pediatric series, 126-patient abstract, 176-patient registry and 306-patient early-death analysis (2019–2023) share program context; no crosswalk, and the 126-patient report is not labelled as an interim cut
OC-SOVIET-ESOPHAGEAL-GRID-1978-1980	ST-00110; ST-KHARITONOVA-OESOPHAGEAL-GRID-22	Esophageal grid (raster) studies published two years apart; authors, sample sizes, and regimens differ, and no accrual dates or identifiers establish continuity
OC-UAMS-GRID-2013-2022	ST-02139; ST-NCT03139318	The single participant in the pediatric osteosarcoma trial closely matches a 17-year-old osteosarcoma case in the 80-patient cohort, which does not cite the NCT identifier; probable overlap
OC-US-NY-HISTORICAL-GRID-1950-1957	ST-00006; ST-00009; ST-00011; ST-00031	Overlapping early New York program context and rare-site signals; no shared identifier, update statement or regimen crosswalk
OC-MAYO-MBRT-2023-2026	ST-MAYO-MBRT-FIRST-2; ST-MAYO-MBRT-MELANOMA; ST-V3-036	The 36-patient orthovoltage MBRT report explicitly follows the two-patient commissioning report; the melanoma case shares program and period. No participant crosswalk

One imaging report described 25 LEAD-treated participants: 21 from the LEAD trial and four BLaStM trial cases selected because of positive endpoint biopsies [20]. Both investigations appear in the evidence map, but the selected cases provide no BLaStM denominator, failure incidence, or separable trial-specific estimate, and the combined imaging findings were not assigned to either trial as independent clinical outcomes. The number of represented investigations therefore exceeds the number of independent, separable clinical-effect estimates.

Source Discrepancies Affecting Interpretation

Discrepancies between or within primary sources were preserved rather than resolved by inference (Supplementary Appendix, section S7; link provided in the Appendix). Accurate transcription alone is insufficient when primary source labels or analysis populations disagree; the main text uses only those portions that can be interpreted without assigning an unsupported denominator or endpoint.

Further conflicts are retained in the report-level records, and the affected quantities are withheld from any aggregate calculation. Pro-Grid (SCR-01951) has discordant response counts between table and text. SCR-01814 reports 52 response categories for a stated imaging set of 51 courses. V3-NEW-022 reports no complete response in Results but two in Discussion, with discrepant dose and toxicity descriptions. An early on-treatment shrinkage count in SCR-01833 differs between Results and Discussion. The LITE SABR packet (SCR-01517) combines a final abstract with an earlier full preprint whose tumor volume summary differs; version-specific values are not combined. The mean complete shrinkage claim in SCR-01198 conflicts with its listed partial response.

Historical tumor classification also requires qualification. SCR-06514 quotes a biopsy diagnosis of “malignant hemangioblastoma” [165]; it is retained based on source-reported malignancy, but this historical label cannot establish a contemporary malignant diagnosis without pathological reassessment and does not underpin malignancy-specific conclusions. SCR-01536 includes one aggressive desmoid tumor in an 11-person cohort, so its nonseparable outcomes are not malignant-only estimates. The pathology boundary restriction in the main article under Quantitative Compatibility and Restricted Syntheses removes both reports.

Incremental Benefit and Comparative Certainty

Table 4 separates direct tests of adding SFRT from internal delivery or timing comparisons and from historical treatment context comparisons. Comparisons only against published literature, paired before-and-after measurements, and single-patient sequential observations are excluded. Because these questions differ and some reports contain only ancillary comparison groups, the rows are not a count of randomized or causally interpretable efficacy tests. Companion reports are grouped without adding participants. The table was assembled during manuscript revision; it was not a prospectively specified subgroup analysis, and no pooled estimate was calculated. Main-text examples prioritize randomized allocation, directness, and availability of group-specific results. Result-specific appraisals are given in the main article under Result-specific Appraisal and Certainty.

**Table 4:** Comparative clinical evidence. CR: complete response; EBRT: external-beam radiotherapy; FDG-PET: fluorodeoxyglucose positron-emission tomography; MLC: multileaf collimator; NSCLC: non-small-cell lung cancer; OS: overall survival; PD: progressive disease; PFS: progression-free survival; PR: partial response; ROBINS-I: Risk of Bias in Non-randomized Studies-of Interventions; RT: radiotherapy; SBRT-PATHY: stereotactic body radiotherapy targeting partial tumor hypoxia; SD: stable disease

Investigation and identifiers	Design, regimen, and comparator	Source-reported results	Principal interpretive limit
Bulky malignancy GRID trial [22]; SCR-00521; ST-NCT00765570	Randomized, open label: GRID plus standard RT (n = 9) versus standard RT (n = 3). GRID: one 15 Gy fraction then 15 standard RT treatments; control: 15 standard RT treatments	GRID versus control: CR 1 vs 0; PR 0 vs 1; SD 6 vs 2; PD 1 vs 0; unknown 1 vs 0. Registry study flow completion 7/9 vs 3/3 (not completion of every prescribed fraction). Zero grade 3–5 protocol-related events posted in each arm	Terminated for slow accrual; one GRID response unknown; no common response time point. Zero posted events do not show safety equivalence. No precise comparative benefit can be inferred
Preoperative rectal irradiation [23]; SCR-00122; ST-00122	Abstract describes randomized allocation of 154 patients: preoperative irradiation plus surgery (n = 76) versus surgery alone (n = 78). Irradiation was open field 4–5 Gy per fraction to 20–25 Gy or GRID 10 Gy per fraction to 50 Gy; surgery 3–5 days later	More than threefold lower postoperative recurrence with combined treatment; no increase in postoperative complications or deaths. No event counts, recurrence time point, or results by irradiation technique	Compares a mixed preoperative RT strategy with surgery and does not isolate GRID. The qualitative recurrence statement is not objective response and yields no GRID-specific effect
SBRT-PATHY in bulky unresectable NSCLC [20–22]; SCR-01169 with SCR-02588, SCR-02619; ST-01169	Nonrandomized three-group comparison: PATHY (n = 20), systemic therapy (n = 20), palliative RT 30 Gy in 10 fractions (n = 20). PATHY targeted a CT/FDG-PET-defined hypoxic segment, 10–12 Gy in 1–3 fractions to the 70% isodose; nine initially single-fraction patients received an adaptive further 10 Gy	Bulky-tumor CR/PR 19/20, 4/20, 4/20; symptom control 16/20, 3/20, 5/20. One-year OS 75%, 60%, 20% (global p = 0.099); one-year PFS 60%, 15%, 0%. Median follow-up 13 months	Assignment depended on age, comorbidity, performance status, and patient choice; co-interventions and prior therapy differ. The 95% figure is CR/PR, not durable control. Symptom definition and timing insufficient; survival-summary inconsistencies, and confounding preclude causal superiority. Companion abstracts add no participants
Cerrobend versus MLC GRID [27]; SCR-00676; ST-00676	Retrospective sequential-era cohorts: Cerrobend (n = 39; 2003–2005) versus MLC (n = 40; 2005–2008). Median GRID dose 15 Gy; 31/39 and 32/40 also received EBRT (median 35 vs 42.5 Gy)	Symptom response: pain 75% vs 74%; mass effect 67% vs 73%. Imaging results (Cerrobend vs MLC): CR 1 vs 3; PR 4 vs 3; SD 12 vs 13; PD 3 vs 1; no follow-up imaging 19 vs 20. Grade 3–4 dermatitis: acute 2 vs 6; late 0 vs 3	Both arms received GRID. Temporal allocation, unequal EBRT dose, and missing imaging in about half limit comparison. Similar symptom rates do not establish noninferiority; toxicity categories and windows must not be summed to count affected patients
Immune-timing partial-tumor irradiation [29,30]; SCR-01789, SCR-02604; ST-NCT04168320	Prospective nonrandomized proof-of-principle comparison of treatment at immune activity peaks versus troughs; expanded journal abstract reports 26 patients	Combined arms: bystander response 77%, abscopal response 41%, 41% alive at median follow-up of 50 months. Timing reported to affect outcomes, but no arm-specific estimates are accessible. The interim conference report (22 patients, 11 per arm) does not identify which numbered arm was peak or trough	Internal timing comparison, not SFRT versus standard RT; the timing effect cannot be quantified. The interim report belongs to the same cohort. Possible overlap with other KABEG studies
Historical esophageal raster versus open field [28]; SCR-00110; ST-00110	Nonrandomized comparative author abstract: raster (n = 31) vs open-field rotation gamma therapy (n = 29). Dose and allocation not reported	Qualitatively fewer and milder esophageal, cardiovascular, and pulmonary reactions with raster; immediate results described as not inferior. No numerical group outcomes or assessment time	No noninferiority margin or test; abstract-only qualitative evidence; possible overlap with another historical esophageal cohort
Historical head and neck reaction comparison [31]; SCR-00031; ST-00031	Retrospective GRID series (n = 48; skin data 37, mucosal data 33) compared with earlier open-portal patients (13 lower dose, 16 higher dose). Exposure units differ (GRID in-air roentgens; open field includes backscatter)	At broadly comparable skin reactions, author-defined mucosal reactions were more intense or prolonged with GRID: second-degree epithelitis or worse 16/20 vs 3/13 (lower dose) and 12/13 vs 7/16 (higher dose). Bilateral second-degree epithelitis in 11/33 GRID patients; not observed with open portal	Reaction and dose-effect study, not a tumor control comparison. Different exposures, nonconcurrent groups, and historical grading prevent conversion to modern grade 3 events. Table and text intensity wording differ; original terminology retained
Historical pancreatic RT comparison [32]; SCR-00097; ST-00097	Author abstract: RT (n = 60) vs symptomatic treatment (n = 10); several irradiation approaches, including grid gamma therapy, 150–200 rad per fraction to 5,000–8,000 rad	Treatment reported ineffective in 31.7%, without defined endpoint, denominator, or arm attribution. No separable GRID-versus-control result	GRID subset and outcome allocation unavailable; comparative context only, with no interpretable SFRT effect or response rate
Historical laryngeal metastasis multimodal comparison [34]; SCR-02926; ST-MOROZOV-LARYNX-METASTASES-52	Indexed abstract: 52 patients; grid telegamma treatment (n = 23), surgery plus RT (n = 16), chemotherapy plus RT (n = 13); translated sequence wording ambiguous	Source term “recovery”: three-year 13/23, 10/16, 5/13; five-year 9/23 and 8/16 for the first two groups.	Unclear allocation, overlapping indications and undefined “recovery”; not interpretable as OS, disease-free survival or local control. Full text unavailable; comparative context only
Preliminary immune-timing comparison [33]; SCR-01198; ST-01198	Prospective nonrandomized sequential series: first four patients treated on a favorable immune-cycle day, next four on an unfavorable day	Bystander response 4/4 vs 2/4; abscopal response 3/4 vs 1/4; symptom relief 4/4 vs 3/4. Median follow-up 11.8 months	Different tumor types and prior treatment; sequential allocation; no conventional-RT comparator; unresolved mean-shrinkage inconsistency; possible KABEG overlap. Serious risk of bias (ROBINS-I) for all three results
Historical heterogeneous GRID series with ancillary lung comparisons [65]; SCR-00037; ST-00037	Series of 69 patients (75 treatments) including 21 lung patients and ancillary non-GRID or untreated lung comparisons	Lung patients had clinical improvement with or without radiographic change; survival summaries use differing origins and survivor or decedent subsets	Ancillary comparisons do not establish exchangeable populations or a common survival estimand; predominantly descriptive, and no comparative effect is computed
Advanced neck disease with surgery-selected groups [88]; SCR-00451; ST-KY-GRID-HNC-1995-2002	Both groups received GRID and further irradiation: one pathway (n = 14) and a surgery-containing pathway (n = 13), with different selection and follow-up	Group 2 overall survival 8/13 at reporting; disease-specific survival reported as a separate endpoint	Operability and treatment pathways differ; does not test the incremental effect of GRID. Disease-specific survival is not substituted for OS

The most direct randomized comparison of GRID added to standard radiotherapy versus standard radiotherapy alone was terminated after enrolling 12 participants [22]. Among the nine participants assigned to GRID, one complete response, no partial responses, six instances of stable disease, one instance of progressive disease, and one unknown response were recorded. Among the three controls, one partial response and two instances of stable disease were recorded. Using all randomized participants as the denominator, documented complete or partial responses occurred in 1/9 versus 1/3 participants, corresponding to an RR of 0.33 (95% CI = 0.03-3.84). No common response assessment time point was reported. Classifying the unknown GRID outcome as a response in a sensitivity analysis did not resolve the substantial imprecision. The response result was judged at high risk of bias using RoB 2 because of missing outcome information, with additional concerns arising from incomplete reporting of trial methods.

The historical rectal cancer trial randomized 154 patients to preoperative irradiation followed by surgery or surgery alone [23]. The irradiation group included patients treated with either GRID or open-field radiotherapy. The abstract reported lower postoperative recurrence but provided insufficient absolute event counts, follow-up details, or separately extractable GRID outcomes. This result was judged at high risk of bias and concerned a mixed preoperative irradiation strategy; it could not isolate the incremental effect of GRID.

A nonrandomized three-arm study enrolled adults with unresectable bulky T4 non-small-cell lung cancer who were considered unsuitable for radical chemoradiotherapy [24]. The study compared stereotactic body radiotherapy-based partial-tumor irradiation targeting putatively hypoxic regions (SBRT-PATHY), systemic therapy, and palliative radiotherapy delivered as 30 Gy in 10 fractions, with 20 patients in each group. Complete or partial responses occurred in 19/20, 4/20, and 4/20 patients, respectively; symptom control was reported in 16/20, 3/20, and 5/20, respectively. Compared with palliative radiotherapy, the unadjusted RR was 4.75 (95% CI = 1.97-11.48) for tumor response and 3.20 (95% CI = 1.45-7.05) for symptom control. However, treatment was allocated by the tumor board based on age, comorbidity, performance status, and patient preference, leaving these associations susceptible to substantial confounding. Common response assessment timing was unclear, and the magnitude and duration of benefit required to define symptom control were unspecified. Conflicting descriptions of median survival and one-year survival precluded a reliable assessment of the survival effect. Companion abstracts reported on the same investigation [25,26].

The comparison of Cerrobend-block and multileaf-collimator GRID radiotherapy evaluated delivery techniques rather than the incremental benefit of adding SFRT [27]. Complete or partial pain relief was documented in 18/24 and 20/27 patients with pain at baseline in the respective groups; pain outcomes were missing for five and three patients. Follow-up imaging was unavailable for 19/39 and 20/40 participants, respectively. Sequential treatment periods, differences in subsequent radiotherapy doses, and short follow-up limited the comparison. Both pain and radiological response results were judged at serious risk of bias, and the study did not establish equivalence or noninferiority.

Table 5 summarizes the four focused certainty assessments.

**Table 5:** Focused comparative questions and certainty of evidence. Counts retain the source populations. Each question draws on one investigation; the two PATHY questions use the same investigation. Risk ratios are unadjusted descriptive contrasts; their confidence intervals describe sampling uncertainty only. GRID: grid-pattern irradiation; PATHY: partial-tumor irradiation targeting hypoxic regions; MLC: multileaf collimator; NSCLC: non-small-cell lung cancer; CI: confidence interval

Clinical question	Observed result	Main limitations	GRADE certainty
Does adding GRID improve objective response at comparable follow-up? [22]	Documented response: 1/9 versus 1/3; risk = ratio 0.33 (95% CI = 0.03-3.84)	High risk of bias, one unknown GRID outcome, no common landmark, and extreme imprecision	Very low
In bulky unresectable NSCLC, does PATHY improve tumor response versus 30 Gy/10 fractions? [24]	Complete or partial response: 19/20 versus 4/20; risk ratio = 4.75 (95% CI = 1.97-11.48)	Serious confounding and uncertain comparability of response timing	Very low
In bulky unresectable NSCLC, does PATHY improve clinically meaningful palliation at defined follow-up? [24]	Symptom control: 16/20 versus 5/20; risk ratio = 3.20 (95% CI = 1.45-7.05)	Serious confounding and subjective assessment; response magnitude, duration, and common timing undefined	Very low
Does Cerrobend versus MLC GRID change pain relief? [27]	Relief documented in 18/24 versus 20/27 initially symptomatic patients; five versus three outcomes missing	Serious confounding, incomplete ascertainment, and imprecision	Very low

The partial-tumor contrasts had CIs excluding the null, but those intervals could not address nonrandom allocation or whether assessment captured comparable, clinically meaningful benefit. Certainty was very low for each defined question. These ratings do not constitute a field-wide rating of every descriptive observation.

Other comparisons evaluated immune timing, delivery methods, or historical combined regimens [28-34]. An additional 32-patient PATHY conference report described bystander and abscopal responses but had possible overlap with other KABEG cohorts; it did not add an independent comparative estimate [35]. In an eight-patient timing study, the first four patients treated during a favorable immune phase were compared with four treated during an unfavorable phase [33]. Bystander, abscopal, and symptom outcomes were more frequent in the first group, but tumor types and prior treatments differed, allocation was sequential, and no common assessment landmark was established. Outcome-specific ROBINS-I judgments were serious. Possible overlap with later institutional reports limited independent corroboration. These comparisons answer different questions from whether SFRT improves otherwise comparable standard care (Table 4).

Result-specific Appraisal and Certainty

RoB 2 used the August 22, 2019, effect-of-assignment variant and ROBINS-I the finalized 2016 instrument with a defined target trial. Confounding was assessed in relation to each outcome rather than as a single whole-study label, and no information, unclear reporting, and demonstrated bias were kept distinct. High risk in the sparse randomized sources does not imply misconduct; it reflects material dependence on missing outcomes and incomplete reporting. In the PATHY comparison, the adjusted cancer-specific survival model does not provide adjusted effects for response, symptom control, or overall survival. Later usual care was not automatically treated as a protocol deviation under an assignment estimand (Table 6).

**Table 6:** Result-specific risk-of-bias judgments. ROB 2: Cochrane risk-of-bias tool for randomized trials; ROBINS-I: Risk of Bias in Non-randomized Studies-of Interventions

Result	Tool	Judgment	Principal reason
R2-GRID-response (NCT00765570 [22])	RoB 2	High	Missing-outcome domain, no-information branch; an information-limited judgment about the available result, not an allegation of misconduct
R2-rectal-recurrence [23]	RoB 2	High	As above
RI-PATHY-response [24]	ROBINS-I	Serious	Uncontrolled confounding; further outcome-specific concerns about measurement or missing data
RI-PATHY-symptom_control [24]	ROBINS-I	Serious	As above
RI-PATHY-one_year_OS [24]	ROBINS-I	Serious	As above
RI-Neuner-pain_response (Cerrobend vs MLC [27])	ROBINS-I	Serious	As above
RI-Neuner-imaging_response [27]	ROBINS-I	Serious	As above
RI-Neuner-severe_dermatitis [27]	ROBINS-I	Serious	As above
Preliminary timing comparison [33]: bystander response, abscopal response, symptom relief	ROBINS-I	Serious (each)	Histology, prior treatment, and treatment sequence unadjusted; unknown measurement or analysis plan information retained as unknown, not as demonstrated bias

Four questions draw on three investigations; both PATHY questions use the same comparison. Each question retains its population, comparator, endpoint, and required follow-up. No certainty rating is assigned to SFRT as a whole, to the inseparable rectal GRID effect, to conflicting survival summaries, or to a participant-level severe toxicity effect that cannot be defined from reported event counts (Table 7).

**Table 7:** Focused GRADE assessments. CR: complete response; GRADE: Grading of Recommendations Assessment, Development and Evaluation; MLC: multileaf collimator; PATHY: partial-tumor irradiation targeting hypoxic regions; PR: partial response; RCT: randomized controlled trial; RT: radiotheraphy; SFRT: spatially fractionated radiotherapy

Question	Evidence	Downgrades	Certainty and interpretation
Objective tumor response at comparable follow-up after adding GRID	1 RCT; 12 participants. GRID: 1 CR, 0 PR of 9; control: 0 CR, 1 PR of 3; one GRID response unknown	Risk of bias −1; inconsistency 0; indirectness −1; imprecision −2; publication bias 0	Very low. The trial is too sparse and incompletely timed to determine benefit or harm
Objective bulky-tumor response at comparable follow-up (PATHY vs palliative RT)	1 nonrandomized study; 40 participants; 19/20 vs 4/20	Risk of bias −2; inconsistency 0; indirectness −1; imprecision 0; publication bias 0	Very low. The large unadjusted association is descriptive and does not establish durable local control or general SFRT superiority
Clinically meaningful symptom relief at a defined, comparable interval (PATHY vs palliative RT)	1 nonrandomized study; 40 participants; 16/20 vs 5/20	Risk of bias −2; inconsistency 0; indirectness −1; imprecision 0; publication bias 0	Very low. The association cannot establish sustained patient benefit free from confounding
Complete or partial pain relief, Cerrobend vs MLC GRID	1 retrospective comparison; 51 baseline-symptomatic patients; documented relief 18/24 vs 20/27 (5/24 and 3/27 not assessed)	Risk of bias −2; inconsistency 0; indirectness 0; imprecision −1; publication bias 0	Very low. No inference of noninferiority or equivalence between delivery methods is justified

Randomized and ROBINS-I target trial bodies started at high certainty and were downgraded once for actual risk of bias. For PATHY, the CIs excluded the null, so small sample size alone did not justify a further imprecision downgrade. The absence of a common response landmark and the undefined magnitude or duration of symptom relief make these endpoints indirect for a time-defined clinical question; differential subjective measurement was addressed under risk of bias. No downgrade was imposed solely because a single study contributed. The full five-domain reasoning and nonupgrade decisions are retained in the archived focused_grade.csv file, available from the corresponding author on reasonable request.

Current JBI updates cover the four recovered full texts and eight newly identified clinical reports, together with abstract-based case-series appraisal of the recovered Mayo early-demise cohort and the new KABEG conference report. Case series and case report items retain their design-specific wording and are not scored. LEAD was appraised with the nine-item legacy quasi-experimental checklist, identified as such rather than relabelled as the revised 2024 instrument. Missing eligibility criteria, nonconsecutive or incomplete inclusion, inconsistent imaging sets, unreported follow-up duration, and source conflicts remain rated as uncertain or unfavorable as appropriate. Clinical data in the modelling and preprint reports were appraised separately from their virtual treatment comparisons. Full item responses, sources, and reasons are retained in the archived JBI files, available from the corresponding author on reasonable request.

Clinical Evidence by Technique and Treatment Pathway

Table 8 summarizes selected clinical investigations by dose geometry, beam type, clinical intent, and subsequent irradiation. These examples distinguish stand-alone irradiation of the index lesion from sequential treatment and incompletely reported pathways; they do not provide comparable response rates across techniques.

**Table 8:** Clinical evidence by technique, treatment intent, and subsequent irradiation. Selected clinical investigations illustrate the treatment pathways underlying the technique-specific synthesis; this table is not a census of the 137 investigations or a comparison of response rates. References in square brackets identify the original clinical reports or posted registry results. Beam type and dose geometry overlap, and no patient totals are summed across rows. Whole-target irradiation means a separate course covering the target, not the lower-dose component within a lattice or core treatment plan. “Not reported” does not establish that no treatment occurred. SFRT: spatially fractionated radiotherapy; LEAD: lattice extreme ablative dose; SBRT: stereotactic body radiotherapy; PATHY: partial-tumor irradiation targeting a hypoxic segment; SCART: stereotactic central/core ablative radiation therapy

Technique and source	Clinical setting and design	Subsequent whole-target irradiation and other treatment	Observation and limits of interpretation
GRID; beam type not specified [22]	Predominantly palliative bulky tumors; randomized registry study, 12 participants	GRID arm: one 15 Gy treatment followed by 15 standard radiation treatments. Control: 15 standard treatments. Concomitant chemotherapy excluded	Documented complete or partial response: 1/9 versus 1/3; one GRID response unknown. No common assessment landmark; very imprecise estimate of adding GRID
GRID; photon [89]	Curative treatment of bulky head-and-neck squamous cell carcinoma; 14-patient series	Yes: GRID followed by definitive intensity-modulated radiotherapy; chemotherapy began with GRID and continued during irradiation	GRID-target control in 11/14 at median follow-up of 19.5 months. Outcomes and acute toxicity describe the combined regimen, without a concurrent control group
GRID; photon [53]	Neoadjuvant treatment of bulky high-risk soft-tissue and osteosarcoma; 26-patient cohort	Yes: GRID before standard conventionally fractionated neoadjuvant radiotherapy and surgery	Pathological-response observations concern the complete preoperative pathway; the incremental contribution of GRID is unestablished
Lattice; photon [42]	Palliative treatment of large extracranial tumors; retrospective cohort, 20 patients	No separate radiation course to the index lesion: the five-fraction lattice regimen was its only irradiation. No concurrent antineoplastic therapy; two-week washout	Three-month response: 11/14 surviving patients assessed; six patients had died. Survivor-based response and short follow-up limit estimates for all treated patients
Lattice; photon, LATTICE_01 [104]	Palliative advanced or metastatic tumors; multicenter series, 30 patients	Yes: vertex irradiation followed within seven days by homogeneous target irradiation; median subsequent prescription 20 Gy in four fractions	Symptom benefit was described after vertex treatment in 3/30 and after completion of irradiation in all patients. Most observations therefore concern sequential treatment
Lattice/LEAD; photon [12]	Definitive prostate treatment; phase I study, 25 men	Yes: intraprostatic lattice boost followed by 76 Gy in 38 fractions to the prostate/proximal seminal vesicles; additional treatment varied by risk	Two biochemical failures at median follow-up of 66 months; one late grade 4 genitourinary event after transurethral resection and sepsis. No randomized estimate of the boost contribution
GRID; proton, Pro-GRID [39]	Palliative bulky invasive cancers; phase I interim cohort, 10 patients	Mixed: eight received subsequent irradiation to the same site (mean 30 Gy); two did not. Systemic therapy was held around SFRT and could subsequently resume	Response categories conflict between aggregate text and the patient table. A grade 3 bowel obstruction occurred after combined irradiation; neither benefit nor harm can be assigned to proton GRID alone
Lattice; proton [116]	Definitive treatment of bulky non-small cell lung cancer; two cases	Yes: proton lattice boost followed by 60 Gy in 30 fractions with concurrent chemotherapy; one patient also received durvalumab	Treated bulky disease remained controlled beyond one year in the reported cases. This describes a combined definitive regimen, not an isolated proton-lattice effect
Minibeam; orthovoltage photons [10]	Urgent symptom relief while preserving subsequent local-treatment options; two cases	Mixed: the Merkel-cell case received conventional irradiation about two months later; the squamous-cell case underwent surgery, after which adjuvant irradiation was not recommended. Both received immunotherapy	Early pain and bleeding improved. Complete clinical response in the Merkel-cell case was documented after subsequent irradiation; the cases do not establish durable minibeam-only control
Partial-tumor SBRT-PATHY; photon [24]	Bulky unresectable non-small cell lung cancer; nonrandomized comparison, 20 patients per group	No subsequent whole-tumor course described for the PATHY group; nine received another adaptive partial-tumor fraction. Conventional palliative irradiation was a separate comparator arm	Response was 19/20 with PATHY versus 4/20 with conventional irradiation. Selection, confounding and outcome-timing limitations prevent causal interpretation
Partial-tumor Particle-PATHY; carbon ions/protons [9]	Strictly palliative recurrent unresectable tumors; 11-patient series including one desmoid tumor	Subsequent whole-tumor irradiation not reported. Five had prior local photon irradiation; two received subsequent immunotherapy after progression	Short-term tumor and symptom observations cannot be separated for malignant tumors alone. These results are not added to an independent particle or GRID/lattice cohort
Central-core SCART; photon [11]	Phase I dose escalation in recurrent/metastatic bulky tumors; patient-level treatment intent not separately specified	Additional irradiation was permitted; two further fractions were described for some patients. Concurrent chemotherapy or immunotherapy was unrestricted. Patient-level whole-target sequence was incompletely reported	Conflicting participant and imaging totals prevent a single unqualified response estimate. Core/peripheral dosing and additional treatment differ from GRID and lattice regimens

GRID: Palliative, Neoadjuvant, and Definitive Treatment

The small randomized GRID study evaluated a boost before standard irradiation, rather than GRID alone [22]. Its comparative results and limitations are detailed in Tables 4-7. Historical palliative series used different endpoints: the 75.7% response reported in an early GRID series combined symptomatic and mass-response assessments and cannot be equated with contemporary objective imaging response [41].

Preoperative and definitive GRID observations also concern combined pathways. In a 26-patient sarcoma cohort, GRID preceded conventional neoadjuvant radiotherapy and surgery; a companion report supplied dosimetric information [53,54]. A cholangiocarcinoma case combined GRID, further irradiation, systemic treatment, and resection [56]. In the 14-patient definitive head-and-neck series, GRID was followed by chemoradiotherapy, with target control reported in 11/14 at a median follow-up of 19.5 months [89]. None isolates the additional contribution of GRID.

The 80-patient GRID cohort recorded 19 acute grade 3 events and six late grade 3 or 5 events, including two fatal carotid blowouts [63]. These are event counts, not counts of distinct affected patients. Completion of additional conventional irradiation was reported in 54/68, but the source alternated between patient and course denominators and included irradiation before or after GRID. Those outcomes therefore cannot be treated as consequences of a uniform GRID-only exposure.

Photon Lattice and LEAD: Palliative Courses and Treatment Boosts

Lattice treatment was delivered both as the only irradiation to an index lesion and before a separate whole-target course. In Iori et al.’s 20-patient palliative cohort, the five-fraction regimen was the lesion’s only irradiation, without concurrent antineoplastic therapy [42]. Complete pain relief was reported in 8/14 patients assessed at three months and 7/10 at six months, with partial relief among the remaining assessed patients. In contrast, LATTICE_01 delivered homogeneous target irradiation within seven days after vertex treatment; most symptom observations followed the combined course [104]. These pathways should not be treated as interchangeable.

The phase I LITE-SABR preprint described 20 treated patients, of whom 13 with 14 tumors had imaging at least 30 days after treatment [36]. All evaluable tumors shrank at the first scan, at a median of 1.8 months, with median volume reduction of 24.4%; this is not a response probability for everyone enrolled. The Lucerne series reported at least 10% volume reduction in 32/40 imaged lesions and rapid relief in 32/37 symptomatic patients, with median follow-up of four months [37]. Its expanded report arose from the same cohort [38].

In the 10-patient prospective breast lattice cohort, nine completed treatment and seven underwent three-month assessment, with four complete and three partial responses [40]. Among seven symptomatic patients, the median pain score fell from 7 to 3. Two relapses followed initial complete responses; actuarial local control was 75% at six months and 50% at one year, despite median follow-up of only 3.8 months. Early regression therefore did not establish durable control.

The phase II lattice registry showed a mixed pain trajectory: mean change on a 0-10 scale was +0.87 at two weeks among 23 respondents, -0.38 at 30 days among 24, and -0.83 at three months among 18 [44]. Changing respondents limit longitudinal interpretation. Local control in 29/32 assessed patients at six months excluded most enrolled participants from that analysis. One of 20 phase I registry participants and two of 66 assessed phase II participants met their respective treatment-related nonhematologic grade 3 or higher toxicity endpoints, through 90 days and six months [44,66]. The final phase I abstract used narrower probable/definite attribution and separately described one possibly related grade 4 event [67]; these results cannot be combined into one incidence.

Definitive LEAD differed from palliative lattice courses. A total of 25 men received a 12 Gy intraprostatic boost followed by 76 Gy in 38 fractions; 14 also received androgen-deprivation therapy [12]. Two biochemical failures occurred over a median follow-up of 66 months, and one late grade 4 genitourinary event followed transurethral resection and sepsis. The registry’s labeling of 66 months as median overall survival was not used as a survival estimate [57]; the earlier conference report described the same cohort [58]. These observations address the combined definitive regimen.

Other lattice applications remained exploratory. A liposarcoma case underwent resection after lattice plus conventional irradiation [55]. A hepatocellular carcinoma series used custom volumetric and enhancement criteria across 12 courses in 11 patients, with frequent co-interventions and short follow-up; it described grade 3 radiation-induced liver disease despite a broader statement limiting acute reactions to grades 0-2 [61]. Additional small series used different prescriptions or incompletely defined symptom measures [47,48].

Proton GRID and Proton Lattice

Particle delivery does not define a single dose geometry or treatment pathway. The early proton-GRID series reported subsequent irradiation in eight of ten patients and subsequent systemic therapy in four; its 80% tumor regression or symptom improvement endpoint was a composite, not an objective response rate [8]. In the palliative Pro-GRID study, eight of ten patients received further irradiation to the same site, and two did not [39]. Its aggregate response categories disagreed with the individual table, and a grade 3 bowel obstruction followed combined irradiation. The two definitive proton-lattice lung cases received conventional chemoradiation after the boost, with durvalumab in one case [116]. Their reported control beyond one year cannot be attributed to proton lattice alone. Particle-PATHY is considered with partial-tumor approaches below, without counting it as a separate additional investigation.

Clinical Minibeam Treatment

The first two orthovoltage-minibeam cases sought rapid symptom relief while preserving later local treatment options [10]. Early pain and bleeding improved, but the Merkel cell case subsequently received conventional irradiation and the squamous cell case underwent surgery; both received immunotherapy. Complete clinical response in the Merkel cell case was documented after the later irradiation. A subsequent minibeam abstract included 36 patients and 46 lesions, with 40 lesions evaluable for response [46]. These observations support further study of that specific technique and setting, not extrapolation from proton, GRID, or lattice outcomes.

Central-Core and Other Partial-Tumor Approaches

The photon SBRT-PATHY comparison treated bulky unresectable lung tumors by targeting a partial volume; conventional palliative irradiation was a separate comparator, not a routine subsequent course in the PATHY arm [24]. Nine PATHY patients received an additional adaptive partial-tumor fraction. The large observed response association remains vulnerable to selection, confounding, and uncertain timing, as detailed in the comparative appraisal. In the palliative Particle-PATHY series, subsequent whole-tumor irradiation was not reported; five patients had prior local irradiation, and two received immunotherapy after progression [9]. Its 11-patient population included a desmoid tumor, and malignant-only outcomes could not be separated. The eight-patient PITH series reported symptom relief at four weeks, but later imaging included both response and progression [43].

SCART should be interpreted separately from patterned GRID or lattice irradiation. The phase I report permitted additional irradiation and unrestricted concurrent systemic therapy, but did not consistently resolve participant totals, imaging denominators, or patient-level treatment sequences [11]. Related SCART reports also contained incompatible totals [59,60]. A 16-patient SCART/lattice abstract described five partial responses plus three instances of stable disease among eight imaged patients as a 100% response rate; only five met its stated partial-response category [49]. These data cannot support an unqualified common response estimate.

Mixed-Technique Cohorts, Harms, and Treatment Completion

Some institutional reports do not support a clean technique-specific outcome estimate. A Mayo sarcoma cohort reported one-year local control of 82% (95% CI = 65%-100%) after 61 courses in 53 patients; conventional irradiation followed 55 courses [50]. Early deaths and an unresolved assessment denominator discrepancy complicated interpretation. A broader, potentially overlapping institutional abstract reported control of 86% at six months and 83% at 12 months, and 14 grade 3 or higher events potentially attributable to any irradiation, including seven fistulae [51]. Cumulative severe toxicity incidence was 7.5% at six months and 13% at 12 months. These findings describe complete treatment courses and must not be transferred to every geometry. Another sarcoma report gave conflicting one-year local recurrence-free survival estimates [52].

A 60-patient retrospective SFRT series reported symptom improvement in 55 at two to four weeks; imaging in 51 showed 17 partial responses, 32 instances of stable disease, and two progressions [45]. A pediatric cohort comprised five children with 10 treatments at eight sites; all developed out-of-field progression despite treated-site observations [62]. Neither supports a common probability of benefit across indications. Historical severe skin and spinal cord injuries remain safety observations without retrospective conversion to modern grades [64,65]. A one-participant lung trial registry recorded serious mediastinal hemorrhage, myocardial infarction, and one death, without sufficient grading or attribution to classify these as SFRT-caused events [68].

All 20 phase I LITE-SABR patients and all 20 patients in Iori et al.’s cohort completed their reported regimens [36,42]. Other reports described an omitted final fraction and a second course stopped after three fractions [69,70]. The phase II registry’s 66/70 “Overall Study” completion milestone did not establish completion of every prescribed fraction [44]. A Mayo abstract recorded 30 deaths within 30 days and 97 within 120 days among 306 treated patients [71]. These are deaths after treatment, not treatment-attributable fatalities; they complicate assessment restricted to survivors. Harms and completion therefore require their original technique, treatment sequence, denominator, and observation window.

Quantitative Compatibility and Restricted Syntheses

No proposed pool provided a coherent, independently supported estimate. Even the closely related five-fraction LATTICE trials reported noninterchangeable completion milestones and different severe toxicity observation windows. Other candidate combinations mixed imaging response with volumetric shrinkage, landmark proportions with actuarial control, or pain means with medians and responder categories. Clinical differences persisted even when two reports used the same broad response criterion. The decision not to pool therefore followed question-specific compatibility assessment, rather than a minimum study count.

Descriptive activity and practical delivery observations persisted when analyses were restricted by design, era, source access, and overlap. Stronger clinical conclusions did not emerge. Full-report restriction removed the small randomized GRID registry result and the phase II registry’s mixed pain trajectory; a more favorable appearance among the remaining reports did not increase causal certainty. Prospective or comparative restriction retained progression, nonuniform symptoms, and relapse after initial response. Removing every possible overlap cluster excluded major institutional response and toxicity reports, thereby narrowing the available evidence rather than disproving their findings.

The one representative per cluster analysis also changed which settings were represented. After retrieval of its complete source, the newer pediatric Mayo report ranked ahead of adult sarcoma and broader institutional reports; it supplied no interchangeable adult one-year control or severe toxicity estimate. Across restrictions, the evidence continued to support observations of activity in selected patients, while the probability, duration, incremental benefit, and full harm profile remained uncertain. Excluding the two diagnostic boundary reports removed their inseparable or historically classified observations without changing the principal comparative, response, palliation, or serious harm conclusions.

The executed matrices contain 912 report investigation claim rows and 822 investigation claim rows covering the six clinical claims. These counts reflect the mapping structure, not the number of positive findings or outcome reporting frequency. Contribution records preserve source unit, timing, extractable observation, uncertainty, and report identity. Survival remains contextual and is not substituted for durable local control, defined in this post hoc analysis as control for at least 12 months. No unique participant total is calculated.

The full-report restriction retains only contributions with an actual full primary source; SCR-01010 and SCR-02256 retain full-text labels in the inventory but lacked recoverable primary texts in this pass and are excluded from it. Retained-summary-only records were also ranked below directly accessible primary material when choosing overlap representatives. The prospective or comparative restriction concerns design, not result direction, and the 2010-onward restriction concerns report year, not treatment year. Both overlap restrictions use the declared clusters (Table 3): one removes all clustered studies; the other retains one representative per cluster, chosen by source completeness, design, whole-cohort coverage, recency, and stable identifier, without reference to outcome direction. The pathology boundary restriction excludes SCR-06514 and SCR-01536. Each restriction was applied separately to the final corpus (Table 9).

**Table 9:** Evidence retained under each separately applied restriction.

Restriction	Retained reports	Represented investigations
Complete-source contributions only	90	87
Exclude uncertain malignant-only applicability	149	135
One representative per possible-overlap cluster	120	108
Exclude every possible-overlap cluster	101	94
Prospective or comparative investigations	32	25
Reports published from 2010 onward	121	107

These are source availability counts; a retained investigation need not contribute an interpretable result to every claim. A restriction may remove direct randomized evidence, adverse observations, or an entire clinical setting, and does not automatically increase certainty.

The full-report restriction removes the randomized GRID registry result and the mixed pain trajectory of the phase II registry, so any more favorable appearance among the remaining reports reflects lost information. The prospective or comparative restriction retains progression, nonuniform symptom change, and relapse after initial response. Excluding overlap clusters removes major institutional response and toxicity contributions without disproving them. The outcome-blind representative rule selects the newer pediatric Mayo study over broader adult cohorts; that report cannot stand in for an adult local control or fistula estimate. The pathology restriction (149 reports; 135 investigations) does not change the principal conclusions. The full claim-by-restriction explanation, including adverse or contrary evidence removed, is retained in the archived claim-by-restriction records, available from the corresponding author on reasonable request.

Fifteen candidate pooling questions were reassessed, each with its indication, regimen, endpoint, time point, analysis unit, candidate investigations, independence assessment, and the reason a common estimate was not defensible. Examples include different severe toxicity windows and denominators in the two five-fraction LATTICE trials, nonequivalent completion milestones, mismatched symptom instruments, selected imaging sets, and potentially overlapping cohorts. The decision matrix is retained in the archived pooling_feasibility.csv file, available from the corresponding author on reasonable request. No pooled effect, heterogeneity statistic, or funnel analysis was calculated.

Within-study contrasts were calculated for two investigations from explicit counts. In NCT00765570, documented response was 1/9 with GRID versus 1/3 with control: RR of 0.33 (approximate 95% CI = 0.03-3.84); risk difference of −22.2 percentage points (−69.0 to 20.1). The GRID denominator includes one unknown response, so this is a documented response contrast, not imputation of failure; an unknown-as-response sensitivity analysis is retained in the calculation file. For PATHY versus 30 Gy in 10 fractions in bulky unresectable NSCLC, response was 19/20 versus 4/20 (RR = 4.75, 95% CI = 1.97-11.48; risk difference = 75.0 points, 46.5-87.6), and symptom control 16/20 versus 5/20 (RR = 3.20, 95% CI = 1.45-7.05; risk difference = 55.0 points, 24.3-73.3). RR intervals were calculated on the log scale and risk difference intervals by the Newcombe-Wilson method. They quantify conditional sampling uncertainty only, not confounding, source conflicts, or uncertain timing; neither the large associations nor intervals excluding the null establish causal benefit.

Figure 2 displays these unadjusted within-study comparisons and their 95% CIs without pooling; the two PATHY outcomes arise from the same cohort [22,24].

**Figure 2:**
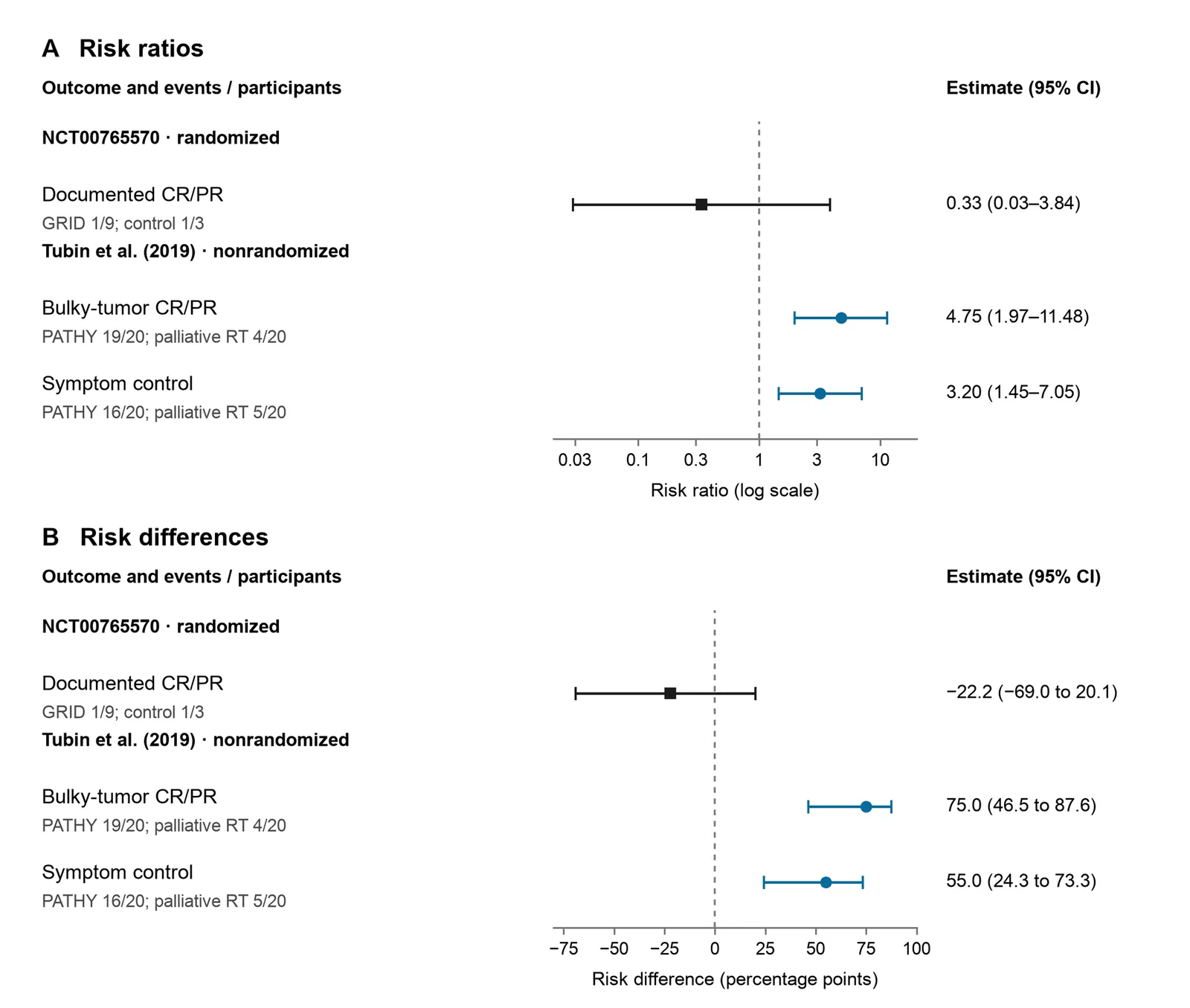
Unpooled within-study comparisons of documented response and symptom control. Panel A shows risk ratios on a logarithmic scale; panel B shows risk differences in percentage points. Symbols indicate point estimates and horizontal lines show 95% confidence intervals (CIs). Dashed vertical lines indicate no difference. Squares represent the randomized GRID comparison; circles represent the nonrandomized PATHY comparison. Counts are events/participants in the intervention and comparator groups. In NCT00765570, GRID followed by standard radiotherapy was compared with standard radiotherapy alone [22]. Its documented complete or partial response (CR/PR) denominator includes one GRID participant with unknown response status. In Tubin et al., SBRT-PATHY was compared with palliative radiotherapy (30 Gy in 10 fractions); both outcome rows use the same patient groups [24]. No pooled estimate or study weights are shown. Risk ratio CIs use the logarithmic method; risk difference CIs combine Wilson score intervals using the Newcombe method without continuity correction. The intervals represent sampling uncertainty conditional on the reported data and assumptions; they do not account for confounding, incomplete measurement or uncertain assessment timing. These descriptive comparisons do not establish causal benefit. Response definitions differ between investigations. RT: radiotherapy; PATHY denotes SBRT-PATHY: stereotactic body radiotherapy-based partial-tumor irradiation targeting putatively hypoxic regions

No RR was calculated for pain relief in the Cerrobend versus MLC comparison [27], because this would have required treating unknown outcomes as failures. Documented responses were 18/24 and 20/27 in the baseline symptomatic populations, with five and three assessments missing; restricting to 18/19 and 20/24 would change the analysis population. No equivalence or noninferiority inference follows.

Discussion

The evidence concerns distinct clinical interventions: photon GRID boosts, photon lattice courses, definitive LEAD boosts, proton GRID or lattice, orthovoltage minibeams, and central-core or other partial-tumor approaches. Their inclusion under SFRT does not establish a shared clinical effect. Outcomes after a boost followed by whole-target irradiation describe a different exposure from outcomes after SFRT as the lesion’s only irradiation. The unresolved question is what each specified technique adds to an otherwise appropriate indication-specific treatment pathway. Direct randomized evidence is extremely limited. Favorable nonrandomized comparisons are vulnerable to prognosis-based selection, differences in accompanying treatment, and uncertain outcome timing. The four focused GRADE assessments were consequently very low certainty, even where the observed association was large. This uncertainty does not establish that SFRT is ineffective; it limits the confidence with which an additional clinical benefit can be claimed.

For palliation, imaging response is an incomplete measure of value. The relevant outcome is relief that occurs soon enough and lasts long enough to matter to the patient, with an acceptable burden of attendance, toxicity, and subsequent care. Several cohorts reported rapid improvement, but the prospective registry’s mixed pain trajectory and shrinking follow-up populations caution against assuming that palliation is uniform. Early death further changes both the opportunity to benefit and the probability of being reassessed. Future comparisons should therefore specify symptom severity and duration, analgesic use, function, and quality of life, and account explicitly for deterioration and death.

Durable local control requires separate evaluation. The prospective breast series illustrates why an initially favorable imaging response cannot substitute for follow-up: relapses occurred after complete responses. Conversely, a one-year actuarial estimate from a small cohort with short median follow-up may rest on very few patients still under observation. Reporting numbers at risk, competing events, and treatment after SFRT would help distinguish sustained local control from survivor selection and the effects of subsequent therapy.

Preoperative and definitive applications require disease-specific comparisons. Pathological necrosis after GRID plus conventional irradiation and surgery is clinically interesting, but the counterfactual is the same overall pathway without the spatially fractionated component. Similarly, biochemical outcomes after a LEAD boost, conventional prostate radiotherapy, and sometimes hormonal treatment cannot establish the boost’s contribution. Trials should connect their technical hypothesis to a clinical endpoint: increased resectability or reduced surgical morbidity, reduced recurrence, sustained symptom relief, or an acceptable late-harm tradeoff. Mechanistic or dosimetric plausibility alone cannot supply that comparison.

Safety is inseparable from anatomy, prior treatment, total dose, co-interventions, and observation time. Fatal carotid events, fistulae, obstruction, and late genitourinary injury deserve explicit reporting alongside cohorts with few recorded complications. Combining them into one toxicity proportion would conceal different exposures and ascertainment. Equally, excluding a retrospective cohort during sensitivity analysis does not erase its observed harm. Prospective toxicity assessment should retain participant-level denominators, distinguish events from affected patients, and specify onset, grading, attribution, and follow-up after subsequent treatment.

This review complements narrower modern-SFRT and LATTICE syntheses by including older experience, multiple techniques, original abstracts, registry results, and explicit report linkage [166,167]. Its structured synthesis demonstrates why access to a full article is not itself a test of validity: restricting to complete publications can remove randomized or unfavorable registry evidence. The useful distinction is whether a result answers the intended clinical question with an appropriate population, comparator, endpoint, and observation period. The focused quantitative contrasts make sampling uncertainty visible, while the source and appraisal records show the other uncertainties that numerical intervals cannot capture.

Important review limitations remain. The protocol was retrospective and unregistered, and the September analyses were specified after the results were known. Retrieval failures were concentrated in older literature, and incomplete abstracts or retained summaries sometimes limited source verification. Missing studies could change the balance of evidence, but their eligibility or direction cannot be inferred from their absence.

The broad eligibility criteria also produced substantial clinical heterogeneity. Historical diagnostic terminology, mixed populations, preprint versions, conflicting source values, possible participant overlap, and inconsistent outcome units required explicit qualification. Restriction analyses test dependence on these features but cannot repair the original studies or prove that the retained evidence is unbiased. Formal comparative certainty assessment was intentionally limited to interpretable questions; it does not substitute for complete reappraisal of every report. The authors confirmed independent human checks while AI assistance was not evaluated as an intervention, and incomplete historical tool records limit reproducibility of that part of the workflow.

The next useful evidence is likely to come from focused, indication-specific comparisons rather than larger collections of heterogeneous response rates. Such studies should define the treatment pathway, document peak and valley doses and geometry, standardize clinical assessment, and retain participants who deteriorate or die in transparent analyses. These priorities align with SFRT trial design recommendations [4] and would allow future syntheses to estimate benefit and harm for a recognizable clinical decision.

## Conclusions

Clinical observations must be interpreted for the specific technique, indication, treatment intent, and complete radiation and systemic treatment pathway. GRID, lattice/LEAD, particle-based treatments, minibeams, and central-core or other partial-tumor approaches cannot be assigned a common efficacy or safety profile from their shared SFRT label. The four assessed comparative questions yielded very low-certainty evidence; the added benefit of the specified interventions remains uncertain. Early regression does not establish durable control, and outcomes after subsequent whole-tumor irradiation cannot be attributed to the SFRT component alone. Technique- and indication-specific comparisons with patient-important endpoints, reproducible dosimetry, and complete follow-up are needed.

## Appendices

The Supplementary Appendix contains the complete database search strings in tabular form, source counts, selection and retrieval records, inventories of excluded and unavailable reports, the historical protocol and September amendments, the source discrepancy assessment (section S7, Supplementary Table S5), and a dated protocol-to-final-methods comparison (section S8, Supplementary Table S6). Reference numbers correspond to those in the main article.
